# Characterization, genomic analysis and preclinical evaluation of the lytic Staphylococcus bacteriophage PSK against methicillin-resistant *Staphylococcus aureus* wound isolate

**DOI:** 10.1186/s12941-025-00783-x

**Published:** 2025-02-28

**Authors:** Abanoub A. Zanaty, Tarek Dishisha, Mohamed Abd El-Gawad El-Sayed-Ahmed, Maha M. Abdel-Fattah, Kawkab A. Ahmed, Karim Abdelkader

**Affiliations:** 1 Department of Microbiology and Immunology, Faculty of Pharmacy, Beni-Suef University, Beni-Suef, 62511 Egypt; 2 Department of Microbiology and Immunology, Faculty of Pharmacy, Misr University for Science and Technology (MUST), Cairo, Egypt; 3 Department of Pharmacology and Toxicology, Faculty of Pharmacy, Beni-Suef University, Beni-Suef, 62511 Egypt; 4 Department of Pathology, Faculty of Veterinary Medicine, Cairo University, Giza, 12211 Egypt

**Keywords:** Bacteriophage, Vancomycin, MRSA, Wound infection, Delayed treatment

## Abstract

**Background:**

The dissemination of multi-drug-resistant bacteria, particularly Methicillin-resistant *Staphylococcus aureus* (MRSA), necessitates exploring new alternatives for their control. Bacteriophages are promising antibiotic alternatives with unique features. Here, we have performed a comprehensive characterization of a newly isolated bacteriophage (PSK) and compared its therapeutic potential with vancomycin in vivo.

**Methods:**

Sewage samples were processed and enriched with the MRSA *S. aureus* SK1 strain in a search for isolation of a lytic bacteriophage. The isolated bacteriophage was assessed in vitro in terms of thermal and pH stability and kinetic parameters using absorption and one step growth curve assays. Moreover, its potential antibacterial activity was evaluated against *S. aureus* SK1 lone and in combination of standard of care antibiotics used for treatment of wound infections. We further analyzed its genome to exclude the presence of any potential toxin or antibiotic resistance genes. Finally, its antibacterial potential and capability to alleviate wound infection were assessed using a murine wound-infection model.

**Results:**

The lytic bacteriophage (PSK) was isolated as a new species of the genus *Rosenblumvirus* with a genome size of 17,571 bp that is free from potential resistance or virulence genes. PSK displays infectivity against 4/10 *S. aureus* strains including two vancomycin-resistant strains. Moreover, it demonstrates favorable infection kinetics of fast adsorption with latent period and burst size of 20 min and 123 PFU/infected cell, respectively. Stability analysis revealed thermal stability up to 60 °C with wide pH range stability (4–11). In vitro, PSK kills *S. aureus* SK1 with multiplicity of infection (MOI) as low as 10^− 4^ with an overall mutation frequency of 2.47 × 10^− 6^ CFU/mL that is further improved when combined with 0.25× MIC of oxacillin, fusidic acid or vancomycin. In vivo, a single dose of PSK in murine wound infection model exhibited a comparable performance to four doses of vancomycin, when treatment started 2 h post-infection. However, when applied 2 days post-infection, PSK demonstrates superior antibacterial activity (up to 4.58 log unit count reduction) and enhances wound closure and tissue healing.

**Conclusion:**

These findings represent PSK as a potential vancomycin alternative effective in treating *S. aureus*- induced wound infections.

**Supplementary Information:**

The online version contains supplementary material available at 10.1186/s12941-025-00783-x.

## Introduction

The widespread dissemination of antibiotic-resistant bacteria may limit the available therapeutic options for their control. This situation heralds the impending post-antibiotic era, particularly given the significant gap in the antibiotic development pipeline. Particularly, methicillin-resistant *Staphylococcus aureus* (MRSA) is a leading nosocomial pathogen that is present as a high-priority pathogen within the WHO list [1–3]. It causes different forms of nosocomial and community acquired infections, including endocarditis, wound abscesses, osteomyelitis, skin and soft tissue infections, and pneumonia, with mortality rates up to 60% and estimated medical costs of 2.5 dollars in the US and European Union [4, 5]. Among them, wound and soft tissue infections are the most predominant forms of MRSA infections [2]. This may be attributed to its natural residence on human skin, which facilitates its introduction through abraded skin, leading to subsequent wound infections.

The treatment of these infections is cumbersome due to MRSA resistance to a wide range of antibiotics, especially β-lactams. Methicillin resistance is mediated by acquiring the mobile element, staphylococcal cassette chromosome mec (*SCCmec*), containing the *MecA* gene that codes for a mutated penicillin-binding protein. This protein displays low binding affinity to β-lactams, rendering them ineffective [6]. Whereas vancomycin is considered the drug of choice for the treatment of MRSA infections, increased reports of vancomycin-resistant *S. aureus* may limit its further prescription [7–9]. Therefore, alternative approaches are urgent to mitigate MRSA infections and to safeguard the remaining effective therapeutic options from being resisted (e.g., vancomycin and linezolid).

Amid the antibiotic resistance crisis, bacteriophage (phage) therapy has emerged as a promising strategy to control the spread of multidrug-resistant pathogens. Phages are bacterial viruses that specifically interact with their bacterial host, leading to their lysis at the end of the lytic cycle. Their potential antibacterial activity has been exploited since their discovery a century ago [10]. Nonetheless, introducing antibiotics has limited their expansion, especially in Western Europe and the USA [11]. Phages have several advantages that set them apart from conventional antibiotics. Their specific interaction with their hosts allows targeted killing without affecting the surrounding flora or selecting resistant mutants. Moreover, their self-replication ensures sustainable delivery with a sufficient concentration at the infection site without the need for booster doses [10]. Also, phages can reach dormant bacterial cells residing within the biofilm matrix through tail-associated depolymerases, making them a possible solution for treating recalcitrant infections [12]. These unique features have fueled research entities to consider phages for subsequent preclinical and clinical evaluations. Several studies have reported the efficacy of the topically applied phages against challenging wound pathogens in different animal models [13–15]. Topical application of bacteriophages could limits adverse immunological responses against phage foreign proteins and septic shock in the case of Gram-negative bacteria. Clinically, phage has been developed up to clinical phase II (https://clinicaltrials.gov/, NCT06319235; Registry date 19/03/2024) in the clinical development pipeline.

Bacteriophages serve not only as alternatives but also as adjuvants for antibiotics. This approach reduces the used antibiotic concentration, thus reducing its possible adverse effects. Moreover, this may reduce the frequency of bacteriophage-insensitive mutants, improving the overall antibacterial activity of both agents [16].

Several studies have explored the antibacterial potential of bacteriophages, both alone and in combination with antibiotics, using murine wound infection models [13, 17, 18]. These studies have shown promising results, including improved wound healing, reduced bacterial counts, and decreased inflammation. However, most inivo experiments involve treating infected animals shortly after wound infection, which does not fully align with real-world clinical scenarios where treatment typically begins at least two days post-infection. This timing difference may lead to overestimated results and may not accurately reflect the true effectiveness of phage therapy in clinical settings. To bridge this research gap, we isolated and conducted a comprehensive in vitro and in silico characterization of the lytic phage vB_SauP_PSK (hereafter referred to as PSK), which targets the wound-associated MRSA strain SK1. Additionally, we performed an vin vivo comparative study to assess PSK as a potential alternative to vancomycin for treating MRSA-induced wounds in a murine model, evaluating its efficacy in both early and delayed treatment scenarios.

## Materials and methods

### Bacterial strain identification, culture conditions, and antibiotic susceptibility

Twenty wound isolates of a presumptively identified strains were collected from patients visiting private laboratories in Cairo, Egypt. Subsequently, the isolates were cultured in 4 mL lysogeny broth (LB; HiMedia Laboratories Pvt. Limited, Mumbai, India) in 15 mL Falcon tubes and incubated at 35 °C for 18 h. Tubes showing turbidity were used for streaking the surfaces of mannitol-salt agar plates (HiMedia Laboratories Pvt. Limited, Mumbai, India), then incubated at 35 °C for 18 h. The plates with yellow colonies were then subjected to a series of conventional biochemical tests for the presumptive identification of *S. aureus* [19]. We used the automated VITEK^®^ (bioMérieux, Marcy l’Etoile, France) to confirm the species-level identity of the strains. The sensitivity of the recovered strains to different antibiotics (oxacillin, cefoxitin vancomycin, linezolid, gentamicin, amoxicillin-clavulanic, fusidic acid, tetracycline, tigecycline, sulphadiazine, and erythromycin) was assessed using the standard Kirby-Bauer disk diffusion method [20].

For the host range analysis, we also included two reference strains (*S. aureus* ATCC43300 and ATCC25923) and four additional previously characterized *S. aureus* clinical strains displaying methicillin (SK10 and SK15) and vancomycin (SK12 and SK30) resistance.

All strains were sub-cultured from their respective glycerol stocks using LB and incubated at 37 °C for 18–20 h. Then, they were streaked over LB agar plates (LB supplemented with 1.5% agar) and incubated under the same conditions.

### Bacteriophage isolation, propagation, and high-titer preparation

Twenty raw sewage samples (50 mL each) were collected from different collecting systems located in Cairo, Egypt, using sterile Falcon tubes. The collected samples were used to isolate lytic phage(s) targeting the MRSA SK1 strain using the enrichment technique as described previously [21] with minor modifications. Briefly, 20–30 mL of sewage samples were centrifuged (5000 ×g at 10 °C for 5 min), filtered using 0.45 μm PES membrane filter (Sigma-Aldrich, UK), and mixed with an equal volume of 2× LB. The sample was then enriched with 200 µl of *S. aureus* SK1 grown till exponential phase (optical density of 0.6) and incubated for 18–20 h at 35 °C. Subsequently, the enriched samples were centrifuged, filtered, and screened for lytic activity using spot-on lawn assay and plaque assays [22].

For purification and high titer preparation, the plaques were collected in 900 µl sterile SM buffer [10 mM Tris-HCl, 10 mM MgSO4, and 100 mM NaCl, pH 7.5], and re-plated at least five times till morphologically similar plaques were obtained. Higher titer stocks were prepared by collecting LB overlay from three semi-confluent plates in 10 mL SM buffer, followed by incubation at 4 °C for 2 h, centrifugation and filtration as before. Finally, phage count was done using plaque assay [22].

### Transmission electron microscopy

The morphology of the isolated phage was examined using transmission electron microscope (TEM) of the pre-filtered high titer phage stock (~ 10^10^ PFU/mL). Briefly, 10 µl of the purified phage was dropped on the carbon-coated copper grid and left for 30 s for drying. Subsequently, negative staining of bacteriophage was done by adding 2% potassium phosphotungstic acid and examined by TEM (JEM-2100, HRTEM, JEOL, Japan).

### Adsorption and one-step growth curve

The infection kinetic parameters of the isolated phage against MRSA SK1 strain were assessed using adsorption and one-step growth curve assays as described previously [23]. The adsorption assay was performed by mixing the purified phage (with a final concentration of 10^4^ PFU/mL) with the exponentially grown MRSA SK1 strain (diluted in LB broth to obtain a final concentration of 10^6^ CFU/mL) to achieve a multiplicity of infection of 0.01. The mixture was incubated at room temperature, then 100 µl samples were withdrawn at 1 min intervals over 5 min. In addition, two samples were taken at 5 and 10 min timepoints. The collected samples were then 10-fold diluted in cold LB broth, centrifuged at 12,000 ×g for 5 min, then supernatants were utilized for counting un-adsorbed phage particles using plaque assay [22]. The experiment was conducted as independent triplicates.

The one-step growth curve was constructed using a change in phage number over 90 min. Briefly, 10 mL of the exponentially grown MRSA SK1 strain (optical density of 0.6) was diluted to 10^6^ CFU/mL in LB, mixed with the purified phage (final concentration of 10^4^ PFU/mL) and incubated for 10 min to facilitate adsorption. The adsorbed phage particles were then pelleted (16,000 ×g for 10 min) and resuspended in 10 mL fresh LB. Subsequently, 100 µl samples were taken at 5 min intervals for 30 min, followed by 10 min for 60 min. Then, the samples were diluted and centrifuged as before, and the supernatants were utilized for phage count. Burst size is determined as the significant increase in phage count/infected cell, whereas the latent period is the time (min) preceding the onset of this change.

### Thermal and pH stability

Thermal and pH stability of the isolated phage were investigated using temperature and pH ranges of 4–70 °C and 4–11, respectively [24]. The thermal stability was assessed by incubating high titer phage stock (10^10^ PFU/mL suspended in SM buffer at pH 7.5) at the specified temperature for 1 h followed by counting the remaining infective phage particles using plaque assay. The pH stability was assessed by 100-fold dilution of high titer phage (10^10^ PFU/mL in SM buffer) in the universal Britton Robinson universal buffer (0.04 M H_3_PO_4_, 0.04 M H_3_BO_3_, 0.04 M CH_3_COOH, and 0.15 M NaCl) adjusted to the test pH range. After 1 h, the phage count at each pH point was detected using plaque assay. All experiments were conducted in three replicates.

### Host range analysis and in vitro antibacterial activity

The host spectrum of the isolated phage was analyzed against a panel of ten *S. aureus* strains (Table 1) using a spot-on lawn assay [25]. Briefly, exponentially grown test strains were individually used to inoculate 4 mL LB soft agar (LB supplemented with 0.6% agar) and mixed well. Next, the seeded soft agar tubes were poured over LB agar plates and left at room temperature until solidified. Finally, 10 µl phage stocks (final concentration of 10^4^ PFU/mL in SM buffer) were dropped over each bacterial lawn and incubated at 35 °C for 18 h. Strains are assigned as sensitive to phage infection by displaying a lysis zone after incubation. Host range results were further confirmed using efficacy of plating (EOP) as described previously [26]. Briefly, the strains showed sensitivity to the PSK stock were spotted with serial dilutions of PSK (prepared in SM buffer) and incubated at 35 °C for plaque counting. Finally, the efficacy of plating was calculated as the phage titer on the test strain was divided by the titer on host bacteria (*S. aureus* SK1).

**Table 1:** **Overview of the*S. aureus*strains used in the current study.** This includes the origin of the used strains, their resistance to different antibiotics and sensitivity to the isolated phage (PSK1)

S. aureus strains	Origin/ source (reference)	Resistance pattern ^a^	Sensitivity to PSK^b^	EOP^c^
SK1	Wound isolate from out-patient in private laboratory, Cairo, Egypt	Oxacillin ^R^, cefoxitin^R^, vancomycin^S^, linezolid ^S^, erythromycin^R^, gentamicin ^R^, amoxycillin-clavulanic ^R^, fusidic acid ^R^, tetracycline^S^, tigecycline ^S^, trimethoprim-sulfamethoxazole ^R^	**+**	1
SK2	Wound isolate from out-patient in private laboratory, Cairo, Egypt	Oxacillin ^S^, cefoxitin^R^, vancomycin ^S^, linezolid ^S^, gentamicin ^S^, amoxycillin-clavulanic ^S^, fusidic acid ^R^, tetracycline ^S^, tigecycline ^S^, trimethoprim-sulfamethoxazole ^S,^ erythromycin^R^	**-**	
SK3	Wound isolate from out-patient in private laboratory, Cairo, Egypt	Oxacillin ^S^, cefoxitin^R^, vancomycin ^S^, linezolid ^S^, gentamicin ^S^, amoxycillin-clavulanic ^S^, fusidic acid ^R^, tetracycline ^S^, tigecycline ^S^, trimethoprim-sulfamethoxazole ^S,^ erythromycin^R^	**-**	
SK4	Wound isolate from out-patient in private laboratory, Cairo, Egypt	Oxacillin ^S^, cefoxitin^R^, vancomycin ^S^, linezolid ^S^, gentamicin ^S^, amoxycillin-clavulanic ^S^, fusidic acid ^R^, tetracycline ^S^, tigecycline ^S^, trimethoprim-sulfamethoxazole ^R,^ erythromycin^R^	**-**	
SK10	Methicillin-resistant strain recovered from pus (unpublished)	Methicillin ^R^, Vancomycin^S^	**-**	
SK15	Methicillin-resistant strain recovered from blood. (unpublished)	Methicillin ^R^, vancomycin^S^	**+**	0.41
SK12	Vancomycin-resistant strain recovered from patient’s wound, Al-Azhar University, Cairo, Egypt. (unpublished)	Methicillin ^R^, vancomycin^R^	**+**	0.92
SK30	Vancomycin-resistant strain recovered from patient’s wound, Al-Azhar University, Cairo, Egypt. (unpublished)	Methicillin ^R^, vancomycin^R^	**+**	0.83
*S. aureus* ATCC43300	Reference strain (https://www.atcc.org/products/43300)	Methicillin ^R^	**-**	
*S. aureus* ATCC25923	Reference strain (https://www.atcc.org/products/25923)	Methicillin-sensitive reference strain for susceptibility testing (CLSI, M2-A9)	**-**	

a: R, antibiotic-resistant, S, antibiotic-sensitive

b: (+) phage-sensitive, (-) phage-resistant

c: Efficacy of plating, ≥ 0.5 (high), 0.1 ≤ EOP < 0.5 (moderate), 0.001 < EOP < 0.1 (low)

The in vitro antibacterial potential of the isolated phage was evaluated against *S. aureus* SK1 strain grown to exponential phase spectrophotometrically as previously described [24]. Briefly, the host strain was mixed with different concentrations of the purified phages to achieve an MOI range of 10^− 4^ − 10 and incubated at 35 °C for 24 h. During incubation, 1 mL samples were collected at 2 h intervals to measure optical density using a spectrophotometer (photometer 4010, Boehringer Mannheim, Ingelheim, Germany). Finally, the measured optical densities were plotted against time. Controls were conducted by incubating plain SM buffer with the bacterial host.

### Bacteriophage-insensitive mutation frequency

The frequency of phage mutation was assessed by subjecting the host strain, *S. aureus* SK1 to high phage titer followed by counting viable colonies. For this, overnight cultured host strain was grown to exponential phase (optical density of 0.6) and then diluted to a final density of 10^6^ CFU/mL. Then, 100 µl of the prepared culture was mixed with an equal volume of the purified phage (or SM buffer as control) to achieve a final MOI of 100 followed by incubation for 10 min at room temperature. Subsequently, the mixture was added to 4 mL soft LB agar, overlayed on LB agar plate and left to dry aseptically. Finally, the viable colonies were counted and compared to control plates (SM-treated culture). Mutation frequency was calculated by dividing the number of viable colonies in the treated culture by those treated with SM buffer. A parallel experiment was conducted by subjecting the prepared host strain to the phage mixed with 0.25× MIC of vancomycin, fusidic acid or oxacillin individually.

**In vitro antibacterial assay**, **MIC assay and synergy analysis**.

MIC analysis of the phage (PSK), oxacillin, vancomycin and fusidic acid was performed using the conventional microdilution broth assay [27]. Briefly, PSK (0.001–10 PFU/mL), oxacillin (2–16 µg/mL), fusidic acid (1–8 µg/mL) and vancomycin (0.25–2 µg/mL) were dispensed into the wells of microtiter plate reader. Subsequently, an exponentially grown *S. aureus* SK1 was added to achieve a final density of 10^6^ CFU/mL. The plates were then incubated at 35 °C for 18 h and examined for growth. MIC was detected as the minimum antibiotic/phage concentration that showed no growth. The obtained MIC values were then used to analyze the potential synergy between phage and each individual antibiotic using checkerboard assay to cover concentration ranges of 0.001–10 PFU/mL and 0.25–2× MIC values respectively [28]. The in vitro antibacterial activity was assessed by treating host strain (with final density of 10^7^ CFU/mL) with the bacteriophage (PSK; to obtain final MOI of 10) in combination with antibiotic (oxacillin, vancomycin or fusidic acid adjusted at final concentration of 0.25× MIC) for 24 h, followed by 10-fold serial dilutions and plating to count the remaining viable cells. Finally, the antibacterial activity was calculated by dividing Log (CFU/mL) of the treated cells by Log (CFU/mL) from the buffer treated cells.

### Bacteriophage genomic extraction and bioinformatic analysis

Bacteriophage genomic extraction from its respective stock (10^12^ PFU/mL) was conducted using the PureLinkTM Microbiome DNA Purification Kit (Invitrogen, USA), which was then utilized to generate libraries with the Nextera XT DNA Library Preparation (Illumina, USA). The Illumina MiniSeq sequenced the genome using a paired-end method (2 × 150 bp). The raw data was quality controlled using Fastp v 0.12.4 [29], and the resultant data was assembled using Unicylcer version 0.4.8 [30]. Assembly quality control was achieved using QUAST 5.0.2 [31]. Subsequently, the RAST online server [32] was exploited for initial annotation of the phage-related contigs. The RAST-predicted functions were further confirmed using Blastx [33], conserved domain database (CDD) [34], InterPro [35] and HHpred [36] online servers. The putative holin topology was analyzed and represented using DeepTMHMM– Predictions [37] Dpofinder was used to detect potential depolymerases [38]. The phage genome was then visualized using the circular genome (CG) viewer online server [39]. The presence of t-RNA and antibiotic resistance genes was screened using tRNAscan-SE [40] and CARD [41] tools. Phage infection cycle was predicted using PhageAI tool [42],. Viral promoters and rho-dependent terminators were detected using MEME [43] and ARNold [44] tools respectively.

Phage taxonomical classification was conducted according to the new roadmap designed by ICTV [45]. Family delineation was analyzed using proteomic-based VipTree against dsDNA bacteriophages (accessed 10th August 2024) [46]. While genus and species demarcations were predicted using Genomic-based tools, Virus Intergenomic Distance Calculator (VIRIDIC) [47] and Virus Classification and Tree Building Online Resource (VICTOR) [48]. Finally a pairwise global genomic alignment was performed with the top ten BLASTn hits using DIGAlign tool [49].

### Murine wound-infection model

Thirty healthy Swiss albino male mice (5–7 weeks old) weighing 20–25 g and procured from Nahda University Beni-Suef Central Animal House, were included in the current study. The animals were housed in well-ventilated cages with temperature optimized at 25 °C using air conditioning and free access to water and antibiotic-free food throughout the experiment. For the experiment, mice were randomly allocated into five experimental groups (*n* = 6) and anesthetized using chloral hydrate (300 mg/kg). Animals were prepared for incision by trimming their dorsal hair followed by complete hair removal using hair removal cream and disinfection with 70% ethyl alcohol. Subsequently, a 6 mm incision was created using a sterile scissor and infected by subcutaneous injection, at the anterior end of the incision, of *S. aureus* SK1 (final density of 10^6^ CFU/mL in sterile PBS buffer pH 7.5) grown to exponential phase. The animals were treated, subcutaneously, with either purified phage (10^8^ PFU/mL in PBS buffer; once) or vancomycin (25 mg/kg/day; divided into two doses for two days). The prepared phage stock was dialyzed by using 3.5 K MWCO Slide-A-Lyzer MINI Dialysis Devices (Thermo Fisher Scientific). The treated groups were subdivided into a group that received the treatment on day 0 (2 h post-infection) and a group that received the treatment 2 days post-infection. Parallel controls were conducted by treating animals with sterile PBS. During the experiment, wound closure rates were calculated on days 0, 2, 4, 6, and 8 using the following equation:

$${\rm{Wound}}\,{\rm{closure}}\,{\rm{rate}}\,{\rm{\% = }}\left[ {{{V0 - Vt} \over {V0}}} \right] \times 100$$

Where V_0_ represents wound size at time zero and V_t_ is the wound size at time t (days). The wounds were photographed at each time interval using a digital camera.

The bacterial count was calculated from the harvested wound tissues on days 2 and 8. The animals were sacrificed, wound tissues were harvested homogenized with PBS buffer, centrifuged (3000 ×g for 5 min), and then serially diluted and plated over mannitol salt agar plates.

### Histopathological analysis of the wound tissues

On day 2 and at the end of the experiment, the mice were euthanized by cervical dislocation. Skin tissue samples were collected from all the mice, fixed in 10% neutral buffered formalin, and processed using the paraffin embedding technique. Tissue sections of 5 μm thickness were prepared and stained with Haematoxylin and Eosin (H&E) at days 2 and 8. Whereas Masson’s Trichrome (MTC) staining was performed to assess collagen deposition at days 8. The samples were examined blindly by a pathologist using a light microscope (BX43, Olympus).

Wound healing parameters were evaluated according to a modified version of the criteria from Bakr et al. (2021) [50]. Briefly, inflammation, granulation tissue formation, and re-epithelialization were assessed blindly in five random microscopic fields per animal (*n* = 3), and scored on a scale of 0 to 3, where (0) represented no changes, and (1), (2), and (3) indicated mild, moderate, and severe changes, respectively. The percentage area of positive MTC staining was visualized and quantified using CellSens Dimensions software (Olympus) [51].

### Ethics statement

All procedures followed the standards set by the Institutional Animal Care and Use Committee (IACUC) of Beni-Suef University. The study adhered to the ARRIVE guidelines and internationally recognized standards for the care and use of laboratory animals, as outlined in the US guidelines (NIH publication #85 − 23, revised 1985). Approval was granted by the Faculty of Pharmacy’s Ethics Committee (Approval no. 022–490).

### Statistical analysis

GraphPad Prism software version 6.1 (GraphPad Software Inc., USA) was used for statistical analysis. For analysis of wound size, statistical significance was assessed using two-way ANOVA test and the multiple comparison was done using Tukey’s multiple comparisons test, the data was expressed as mean ± standard error of means (M ± SEM). For the statistical analysis of the histopathological scores of necrosis and inflammatory cells infiltration, the data expressed as the median and interquartile range (p25-p75). Statistics were carried out by Kruskal Wallis test followed by Dunn test. Finally, Student’s t test was used to test the difference in the antibacterial activity of phage- or vancomycin-treated group compared to PBS treated group. p values of < 0.05 were considered statistically significant.

## Results

### Bacterial identification and antibiotic sensitivity

Four *S. aureus* strains were presumptively identified using conventional biochemical testing and their identity was further confirmed using the automated VITEK’s identification system. Subsequently, oxacillin susceptibility testing was performed using the Kirby-Bauer disk diffusion method to select MRSA strains for further experimental analysis. Only one strain, *S. aureus* SK1 was resistant to oxacillin and cefoxitin, thus assigned as MRSA. Then, we expanded the sensitivity testing to include a panel of antibiotics prescribed to treat *S. aureus* infections (Table 1). The four strains were resistant to fusidic acid and erythromycin. Nonetheless, *S. aureus* SK1 showed additional resistance to amoxicillin-clavulanic, gentamicin and sulphadiazine (Table 1), hence underscoring this strain as a problematic strain refractory to common topically applied antibiotics. Interestingly, all strains were sensitive to vancomycin and linezolid, the last-resort antibiotics against *S. aureus* indicating their efficiency at least in our case.

### Phage vB_SauP_PSK is a podovirus with a favorable kinetic and stability profile

**Fig. 1:**
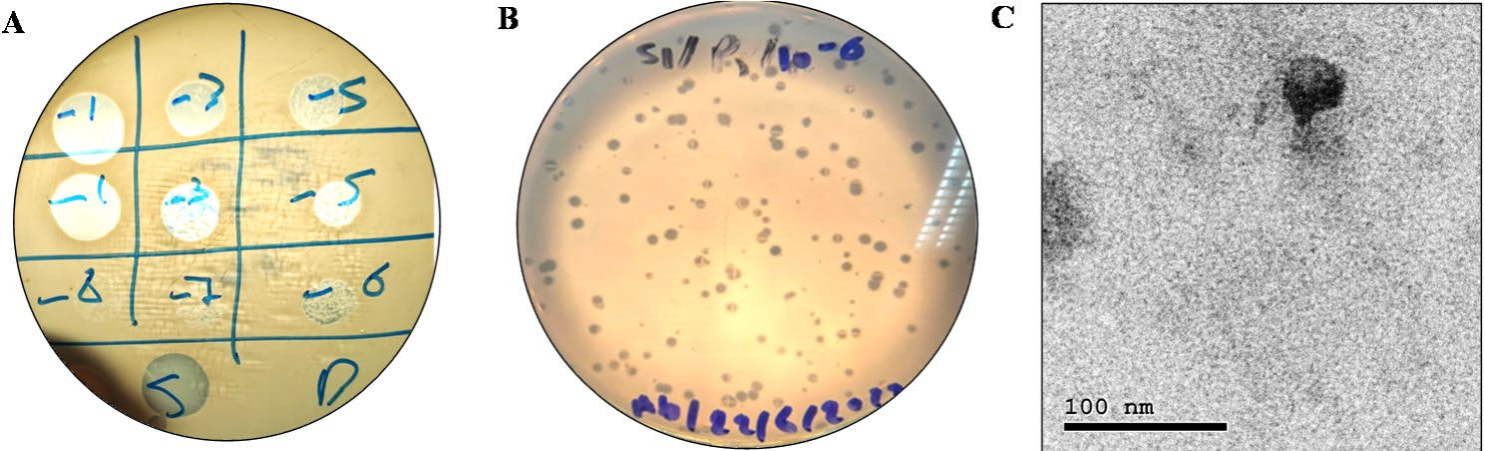
Morphological and cultural characteristics of Staphylococcus phage PSK. (**A**) PSK lytic activity was screened by spotting filter-sterilized enriched sewage over the surface soft LB overlay agar seeded with *S. aureus* SK1. (**B**) Purified plaques were obtained by replating individual plaques at least five times. (**C**) TEM imaging was used for morphological analysis of PSK at 100 nm scale bar

Only MRSA strain, *S. aureus* SK1 was used as a host strain to isolate a suitable phage from raw sewage samples. The samples were collected from the main sewage system that is assembled from the Cairo University Hospital drains, where the *S. aureus* SK1 strain was isolated. Spot-on lawn analysis of one processed sewage sample revealed a lysis zone (Fig. 1A), indicating a potential lytic phage (vB_SauP_PSK, thereafter known as PSK). PSK forms circular and clear plaques with an average diameter of 1.6 mm following double-layer assay (Fig. 1B). TEM analysis of the phage suspension (Final concentration of 10^10^ PFU/mL) showed a short-tailed phage with an icosahedral head, indicating a podovirus morphology, belonging to class *Caudoviricetes* (tailed-phages; Fig. 1C).

**Fig. 2:**
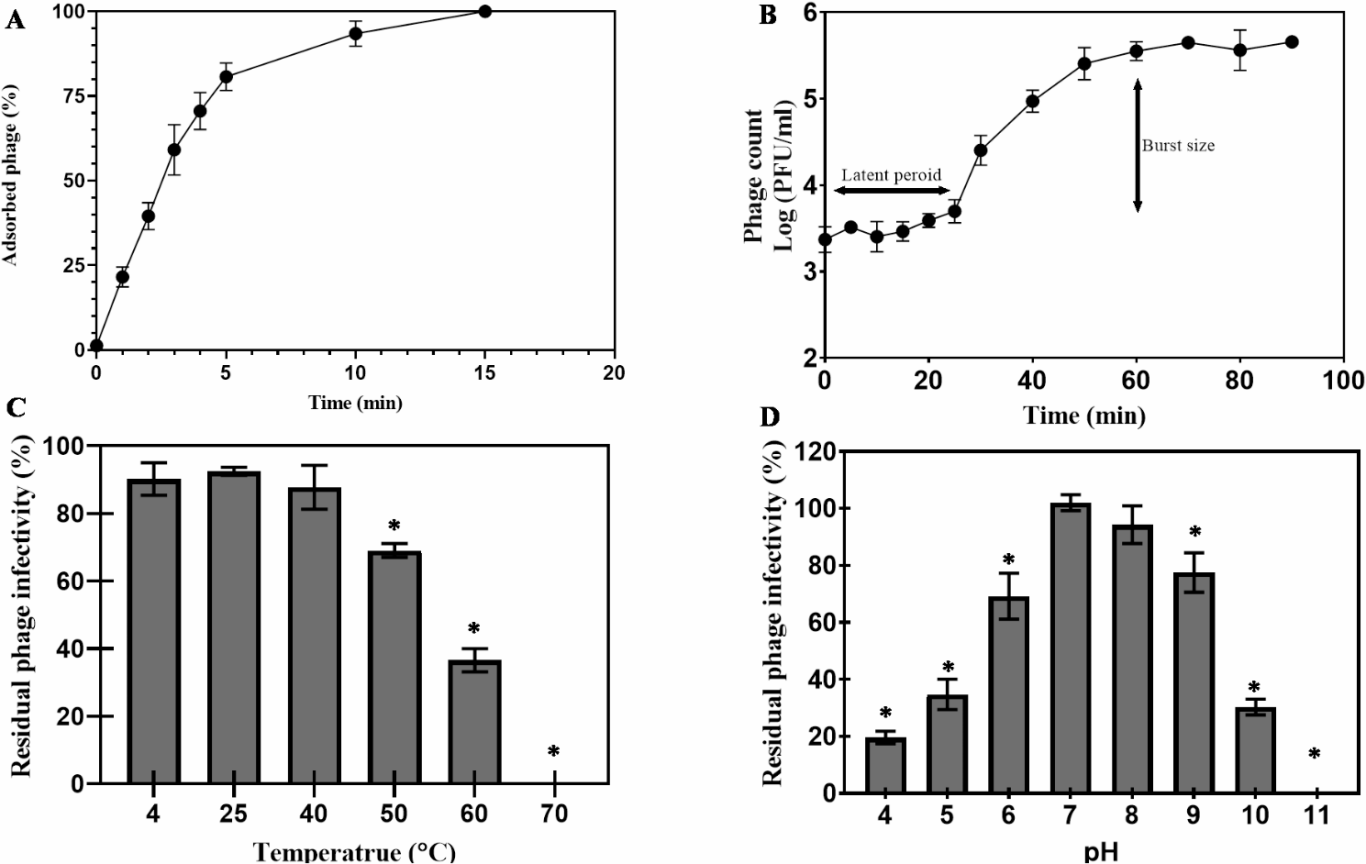
Kinetic and stability evaluation of Staphylococcus phage PSK. (**A**) Adsorption curve was obtained by mixing PSK with its host (*S. aureus* SK1) at MOI of 0.01, then free phages were counted, expressed as percentage and plotted against time (min). (**B**) One-step growth curve was constructed by mixing PSK and its host stains at final MOI of 0.01, then phage count was monitored over 90 min. (**C**) Thermal stability of PSK was assessed by incubating it for 1 h at test temperature, then serially diluted, counted and expressed as percentage. (**D**) pH stability was validated by incubating PSK (10^10^ PFU/mL suspended in SM buffer at pH 7.5) at different pH values (4–11), then infective PSK were counted and expressed as percentage. Each value represents the mean of three replicates ± standard deviation. A paired Student t test was conducted to compare significance between mean values, with a temperature of 4 °C and pH 7 as references (*, *p* < 0.05)

Infection parameters of PSK were investigated using adsorption and one-step growth curve. PSK shows a relatively fast adsorption with more than 75% of PSK particles adsorbed within the first 5 min. While complete adsorption is reached after 15 min (Fig. 2A). The one-step growth curve revealed a latent period of 20 min followed by an increase in phage count by 2 log units (burst size of 123 PFU/ infected cell) reaching its maximum count after 60 min (Fig. 2B).

Stability assessment of PSK showed a wide range of thermal stability (up to 60 °C) with a significant infectivity reduction (*p* < 0.05) to 69.06 ± 2.05 and 36.56 ± 3.43% when exposed to 50 °C and 60 °C respectively (Fig. 2C). Exposing PSK to 70 °C for 1 h eliminated its infectivity (Fig. 2C). In addition, it is stable over the pH range of 4 to 10 with the maximum infectivity observed at pH values 7 and 8, 103 ± 3% and 94.3 ± 6.60%, respectively (Fig. 2D). A slight reduction in PSK infectivity was noted at pH 9 (77.5 ± 6.6; *p* < 0.05) which is further lowered at pH 10 (30.3 ± 2.8%; *p* < 0.05) and completely abolished at pH 11 (Fig. 2D). On the other hand, PSK displays a relatively higher tolerance to the acidic pH values (4–6) that is observed as gradual reduction in the phage infectivity with lowering pH reaching its minimum infectivity at pH 4 (Fig. 2D).

### In silico analysis of the phage genome reveals its suitability for therapeutic application

**Fig. 3:**
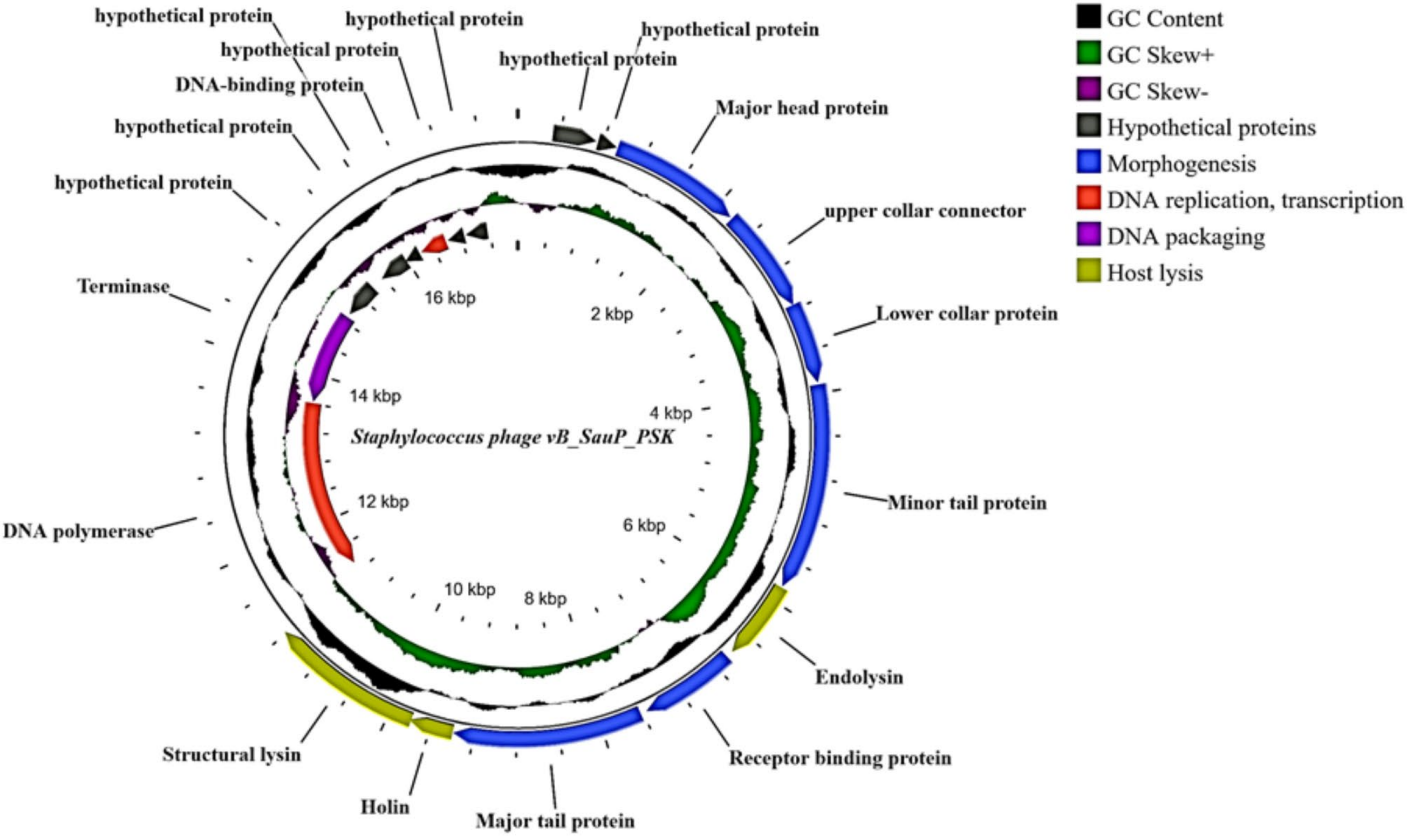
PSK genomic visualization and annotation using CG view tool. Each open reading frame was labeled with its putative function. Functional modules were differently colored as represented in the upper right-handed corner. The arrows represent transcriptional direction. The second and third rings from outside represent GC content and skew respectively

Genomic analysis of PSK revealed a double stranded linear genomic DNA (accession number PQ110032), with a length and GC content of 17,571 bp and 29%, respectively (Fig. 3). The completeness of the sequenced genome was verified by the presence of terminal repeats at both ends (Supplementary Table S1). Genomic annotation predicted 19 open reading frames (ORFs, Supplementary Table S1 and Fig. 3) with different transcriptional directions (distributed on both leading and complementary strands) and starting with ATG codon, except for ORF14, which starts with TTG. The functions of twelve coding sequences (63.16%) were predicted based on the conserved domains detected within the amino acid sequences of their respective protein products (Fig. 3; Table S1 in the supplementary material). The assigned functions categorized ORFs into four different modules including morphogenesis (*n* = 6, 31.5%), DNA replication (*n* = 2, 10.5%), DNA packaging (*n* = 1, 5.26%) and host lysis (*n* = 3, 15.8%) encoding genes. The remaining seven coding sequences showed no homology to any of the identified conserved domains, thus assigned as hypothetical proteins (protein with unknown functions; Fig. 3, Table S1 in the supplementary material). PSK adopted the two-components host lysis system (holin-lysin system). Structural analysis of the lysis proteins revealed a class II holin with two transmembrane domains (Figure S1), and a modular lysin with N-terminal enzymatically active domain (CHAP domain, pfam PF05257) and C-terminal cell binding domain (SH3-5; PF24246). Six putative promoters (Supplementary Table S2) and one putative rho-independent transcription terminator (Supplementary Table S3) were detected throughout the phage genome, whereas no t-RNA or depolymerases were observed.

The utility of PSK for therapeutic application was investigated using a plethora of online servers designed to detect phage lifestyle and occurrence of possible antibiotic resistance or virulence-related genes. The lytic nature of PSK was predicted using a machine learning-based tool (Phage AI) with confidence of 95.51%. This is further corroborated by the absence of any potential lysogeny-related genes (recombinase, excissionase, integrase) upon manual inspection. Furthermore, neither antibiotic resistance nor virulence genes were detected throughout the phage genes, indicating PSK as a possible candidate for phage therapy without worries about disseminating resistance genes amongst the infected bacterial populations.

### The phage PSK represents a new species of the genus *Rosenblumvirus*

**Fig. 4:**
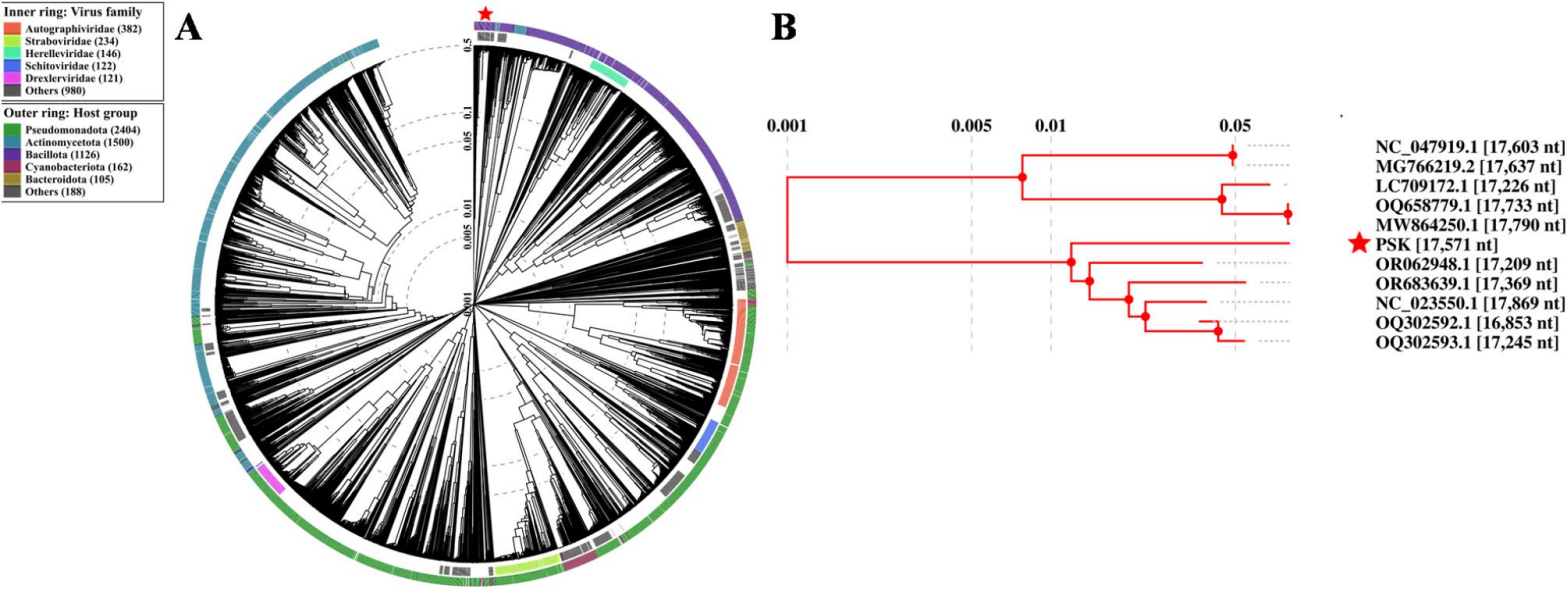
Proteomic tree of PSK genome using VipTree online tool. (**A**) Circular proteomic tree of PSK genome (labeled with star) with genomes of other phages belonging to different taxonomical families. The inner and outer rings represent host group and virus families respectively. (**B**) Rectangular phylogenetic tree of PSK (labeled with red star) with its close relatives based on nucleotide similarity

**Fig. 5:**
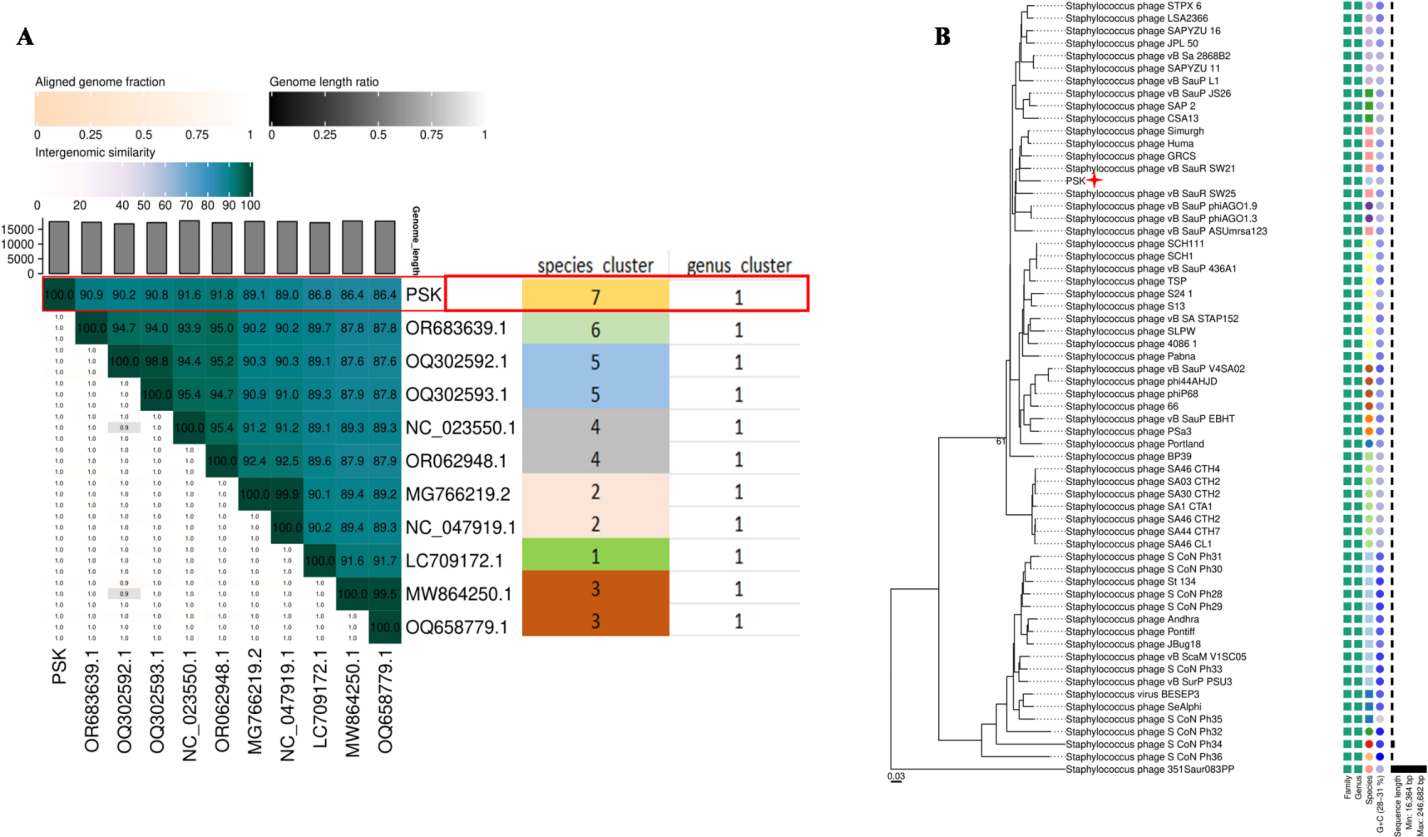
Taxonomical classification of PSK using intergenomic similarity. (**A**) VIRIDC heatmap represents the intergenomic similarity of PSK with top ten BLASTn hits. Per square, the percentage of intergenomic similarity between phages pairs according to the intergenomic similarity scale (on the top). The other scales represent the ration of the aligned genomes lengths and fractions. The differently colored rectangles highlight the different species cluster to which each phage belongs. (**B**) VICTOR genome-based phylogenetic tree using pair-wise nucleotide comparison with other phages belonging to the genus *Rosenblumvirus.* Differently colored circles represent the different species

Taxonomical classification of the PSK was performed following the new guidelines established by ICTV [45]. Therefore, a plethora of online tools were exploited to analyze genomic, intergenomic and proteomic similarities with other homologous phages. Initially, family classification was predicted using the proteome-based clustering tool (VipTree) against > 3000 nucleotide sequence of dsDNA phages. Unfortunately, VipTree couldn’t provide an accurate family demarcation, assigning PSK under unnamed and underrepresented family as Others (Fig. 4). Nonetheless, genomic similarity analysis (BLASTn) displayed high identity levels (up to 94%) with other *S. aureus* infecting phages belonging to genus *Rosenblumvirus* of *Rountreeviridae* family (Supplementary Table S2), making Staphylococcus phage GRCS (NC_023550.1; identity of 93.66 and coverage of 96%) the closet relative. Interestingly, none of the homologous phages showed nucleotide similarity ≥ 95%, proposing PSK as a new species under genus *Rosenblumvirus.* This assumption was further verified by the results of intergenomic similarity analyzing (VIRIDIC) and VICTOR genes clustering tools. VIRIDIC showed maximum intergenomic similarity of 91.8% with Staphylococcus phage 351Saur083PP (accession number OR062948) with no intergenomic sequence similarity exceeding 95% (Fig. 5A). Moreover, VIRIDC and VICTOR tools have clustered PSK1 as different species of the same genus (Fig. 5A and B). Finally, DiGAlign global alignment with the top six homologous phages indicated high similarity and synteny with the aligned sequences except for the genes coding the C-terminus of the receptor binding protein (RBR, *orf8*) and tail fiber (*orf9*) (Supplementary Figure S2).

### The phage PSK possesses strong lytic activity against MRSA with a low resistance frequency

**Fig. 6:**
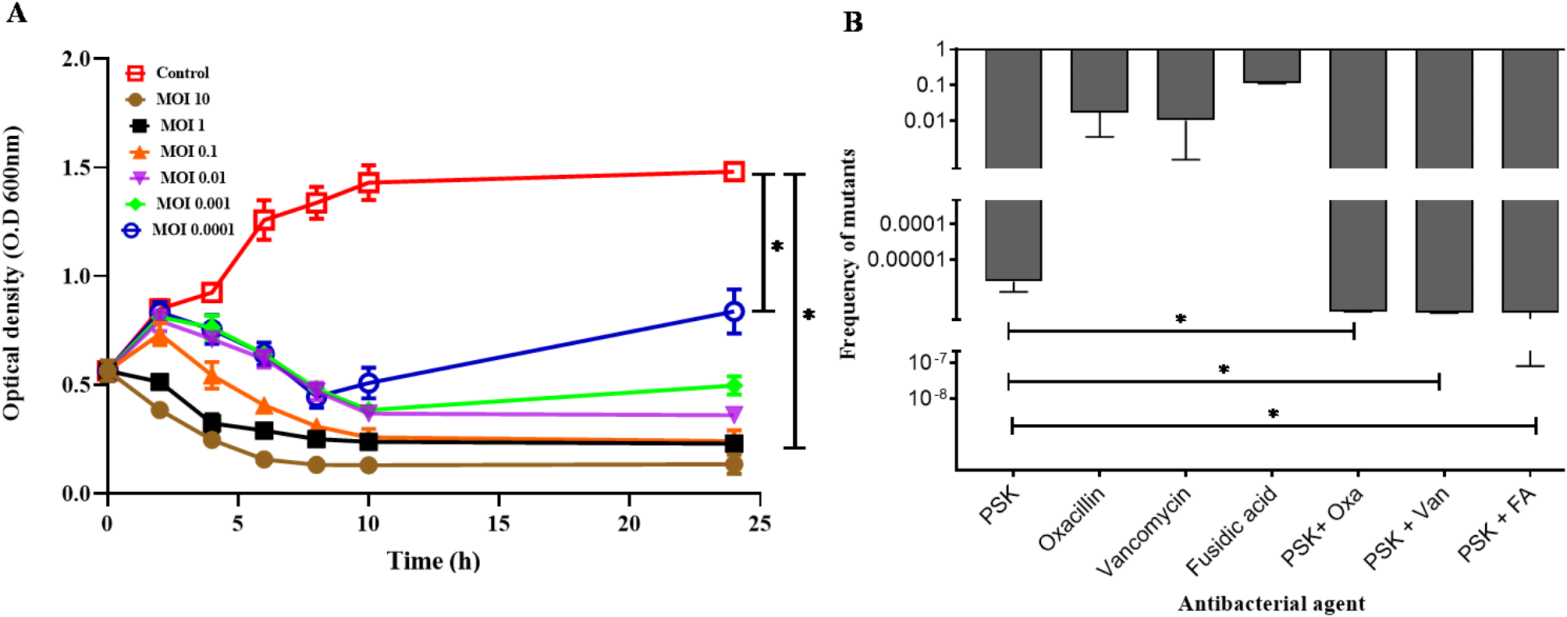
The lytic activity of PSK and mutation frequency against *S. aureus* SK1. (**A**) PSK lytic activity was assessed by nixing it with *S. aureus SK1* at different MOIs (10^− 4^ – 10). Bacterial growth was monitored turbidimetrically over 24 h. (**B**) Mutation frequency of PSK, vancomycin, oxacillin, fusidic and combination of PSK with each of them was assessed against *S. aureus SK1.* The bacterial strain was mixed with PSK (at final MOI of 100) alone or in combination with each antibiotic (final concentration equals 0.25 MIC). Then, viable cells were counted as CFU/mL and dived by the initial bacterial count. All values represent the means ± the standard deviations (SD) of three biological replicates. A paired Student t test was conducted to compare significance between mean values, with PSK alone as a reference (*, *p* < 0.05). FA, fusidic acid; Van, vancomycin; Oxa, oxacillin

Host range analysis of PSK was established against a panel of ten *S. aureus* strains with varying resistance patterns, including vancomycin-resistant strains, *S. aureus* S12 and S30 (Table 1). Four out of the tested strains were sensitive to PSK, including S12 and S30 strains. To exclude any potential lysis from without, EOP was conducted on the strains showing sensitivity to PSK. All sensitive strains showed EOP ≥ 0.5, except for *S. aureus* SK15 (EOP of 0.4; Table 1). Subsequently, we proceeded to investigate the in vitro antibacterial potential of PSK by mixing it with its host strain *S. aureus* SK1 at different MOIs (10^− 4^ – 10), then monitoring bacterial growth turbidimetrically. PSK significantly inhibited (*p* < 0.05) bacterial growth at all tested concentrations (as low as final MOI of 10^− 4^) with the maximum activity observed at MOIs of 10 and 1 (Fig. 6A). Nevertheless, none of the tested MOIs was able to totally eradicate host strain, appeared as survival colonies after subculturing the tested mixtures at 30 h. This indicates a possible emergence of bacteriophage-insensitive mutants (BIM) following prolonged incubation, a common limitation of using phage. We further analyzed the resistance frequency to PSK by mixing it with the host strain at MOI of 100 for 10 min. PSK displays a relatively low resistance frequency of 2.47 × 10^− 6^ ± 1.19 × 10^− 6^ CFU/mL, reinforcing our hypothesis regarding emergence of BIM. To investigate the change in the resistance frequency in case of phage-antibiotic combination, we have re-conducted resistance frequency experiment in presence of PSK–antibiotic combination (adjusted to obtain final concentration equal to 0.25× MIC), and calculated resistance frequency as before, then compared it with those treated with each individual agent alone. Individual antibiotics displayed a significantly higher resistance frequency, 3 × 10^− 2^ ± 2.9 × 10^− 2^, than PSK alone without significant difference between them. Nonetheless, combining PSK with each antibiotic individually, significantly lowered mutation frequency (*p <* 0.05) down to 10^− 8^ (Fig. 6B), irrespective of the used antibiotic. This underscores the advantage of the phage-antibiotic combination in increasing the sensitivity of bacteria to phage infection, thereby mitigating one of the limitations of phage therapy.

### Antibiotics combination does not improve PSK antibacterial activity

**Fig. 7:**
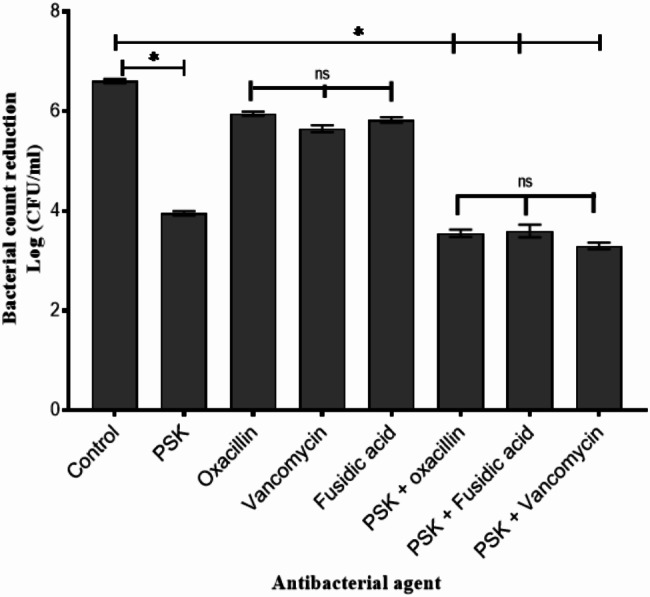
The in vitro antibacterial activity of PSK alone and in combination of oxacillin, vancomycin and fusidic acid. The exponentially grown *S. aureus* SK1cells and adjusted to 10^7^CFU/mL and exposed to either PSK alone (with final MOI of 10) or in combination or oxacillin, vancomycin or fusidic acid at final concentration of 0.25 MIC. The overall antibacterial activity was calculated by dividing Log (CFU/mL) of treated cells by those from un-treated cells. All values represent the means ± the standard deviations (SD) of three biological replicates. A paired Student t test was conducted to compare significance (*, *p* < 0.05) between untreated group and each treated group individually. Statistical significance was assessed using two-way ANOVA test, the multiple comparison was done using Tukey’s multiple comparisons test

Antibiotics have been reported to improve the overall antibacterial activity of phages when used in combination, a growing term coined phage-antibiotic synergy (PAS) [47]. Therefore, we investigated whether combining PSK with the antibiotics prescribed to treat *S. aureus* infections (oxacillin, vancomycin, and fusidic acid) could enhance PSK antibacterial activity. Thus, the conventional checkerboard assay was conducted to evaluate the potential synergy between PSK and other anti-staphylococcus antibiotics (oxacillin, vancomycin, and fusidic acid) against *S. aureus* SK1. The concentration ranges were adjusted to cover 10^− 2^ − 10^2^ PFU/mL for PSK and (0.25× MIC − 4× MIC) for the tested antibiotics. Expectedly, PSK showed no MIC (complete turbidity reduction) within the tested range, up to 10^2^ PFU/mL. This is attributed to the emergence of BIM that are able to regrow in the presence of PSK. On the other hand, oxacillin, fusidic acid, and vancomycin displayed MICs of 8, 4 and 1 µg/mL, respectively, against *S. aureus* SK1. Notably, none of the PSK-antibiotic combinations showed a synergistic outcome when tested against *S. aureus* SK1 by checkerboard assay. To have an in-depth insight into the antibacterial activity of the PSK-antibiotic combination, a bacterial count reduction assay was performed in SM buffer. For this, exponentially grown *S. aureus* SK1 was diluted to 10^7^ CFU/mL and treated with PSK (adjusted to obtain a final MOI of 10), antibiotics (oxacillin, vancomycin, or fusidic acid; diluted to obtain final concentrations of 0.25× MIC), and a combination of both. Alone, PSK has reduced the initial bacterial count by ~ 4.21 log units (*p* < 0.05; Fig. 7), whereas individual antibiotics reduced bacterial count up to 1 log-unit without significant difference between them (*p* > 0.05; Fig. 7). In consistence with the results obtained from the checkerboard assay, PSK-antibiotic combinations showed no improvement in the overall antibacterial activity of PSK when compared to PSK alone (*p* > 0.05; Fig. 7).

### PSK outperforms Vancomycin in delayed treatment of murine wound infection model

**Fig. 8:**
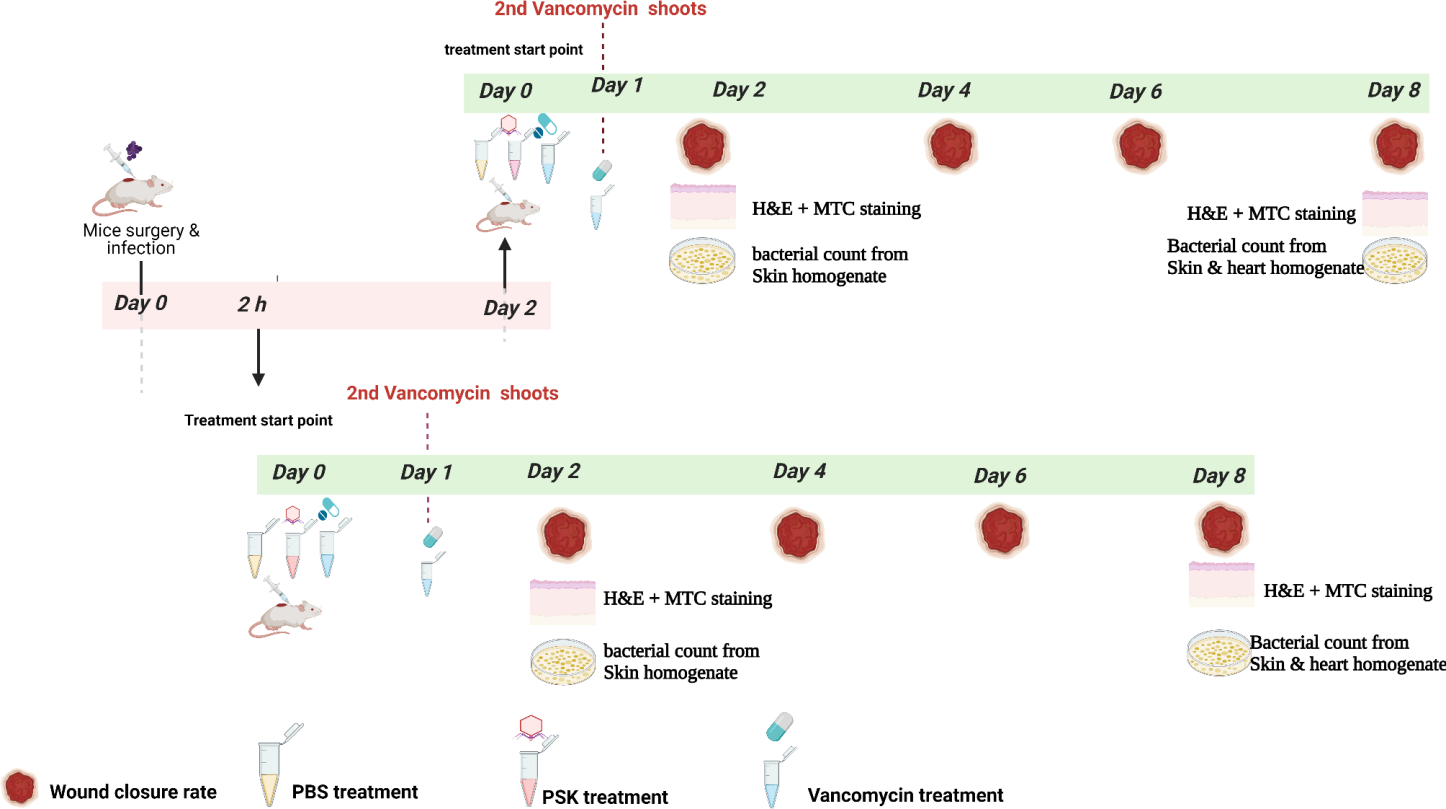
Schematic representation of the therapeutic efficacy workflow for PSK and vancomycin in a murine wound infection model. The wounded mice were challenged with 10^8^ CFU/mL, then treated subcutaneously 2 hpi (the lower panel) or 2 dpi (the upper panel) with PSK vancomycin or PBS. The wound diameter was measured at days 2, 4, 6 and 8. The skin tissues from each group were stained with Hematoxylin and Eosin (H&E) at days 2 and 8 or MTC at day 8 and examined microscopically. Finally, the bacterial count within skin tissues was counted on days 2 and 8

**Fig. 9:**
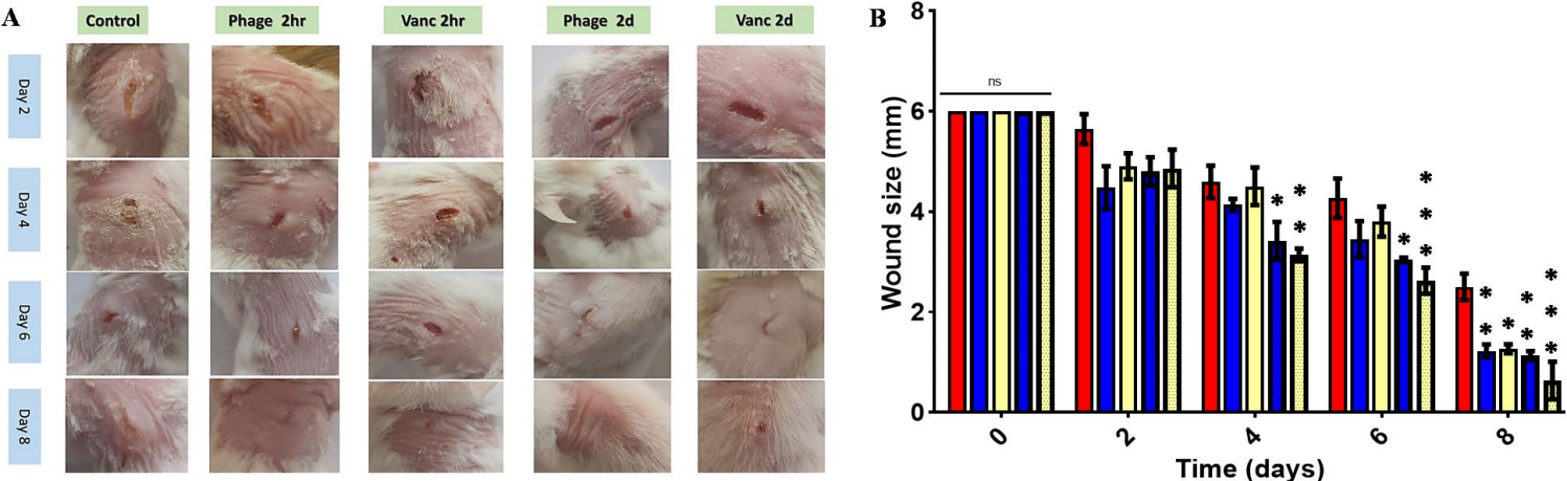
The rate of wound closure in skin excisions during bacterial infection. (**A**) Representative photographs of wounds from control, PSK and vancomycin treated mice 2hpi and 2 dpi. (**B**) Wound size (mm) from each group (*n* = 3) at days 0, 2, 4, 6 and 8. Each value represents mean ± standard error of mean. Statistical significance was assessed using two-way ANOVA test, the multiple comparison was done using Tukey’s multiple comparisons test. *, *p* < 0.05; **, *p* < 0.01; ***, *p* < 0.001

Vancomycin is the standard treatment for MRSA infections because it has a molecular target different form the modified PBP-2a, present in *mecA*-positive MRSA [52]. Fusidic acid and gentamicin are also possible topical options for treating MRSA infections. However, in our study, the host strain, *S. aureus* SK1, was resistant to both topical antibiotics (Table 1). Consequently, we conducted a comparative analysis of PSK and vancomycin in treating wound infections in a murine model, focusing on the effects of early (2 h post-infection, 2 hpi) and delayed (2 days post-infection, 2 dpi) interventions on overall wound healing (Fig. 8). Delayed intervention would provide more time for bacteria to establish more complicated infection and possible dissemination to internal organs (e.g., heart). The establishment of wound infection was verified by the inflamed and suppurative wounds present in all experimental groups (Data not shown). Two-hours post-infection, the groups were either treated with vancomycin (25 mg/kg/day), PSK (final concentration of 10^10^ PFU/mL) or sterile PBS (control group) to establish fast-treatment intervention, then wounds were visualized two days after intervention for a period of 8 days (Fig. 9A). The wounds among the control group showed a slow and gradual closure rate with the minimum wound diameter observed on day 8 of 2.5 ± 2.65 mm (Fig. 9B). On the other hand, early treatment (2hpi) with either vancomycin (4 divided doses) or PSK (one dose) has shown a comparable improvement (*p* < 0.05) in the overall extent of the wound closure to be 1.22 ± 0.133 and 1.26 ± 0.088 mm at day 8, respectively (Fig. 9). However, neither of both treatments has completely shown complete wound closure. Unexpectedly, delayed treatment with either vancomycin or PSK displayed a faster wound closure rate started at day 4, with a relatively higher extent for PSK that successfully showed almost complete wound closure at day 8 (0.63 ± 0.36 mm; Fig. 9).

**Fig. 10:**
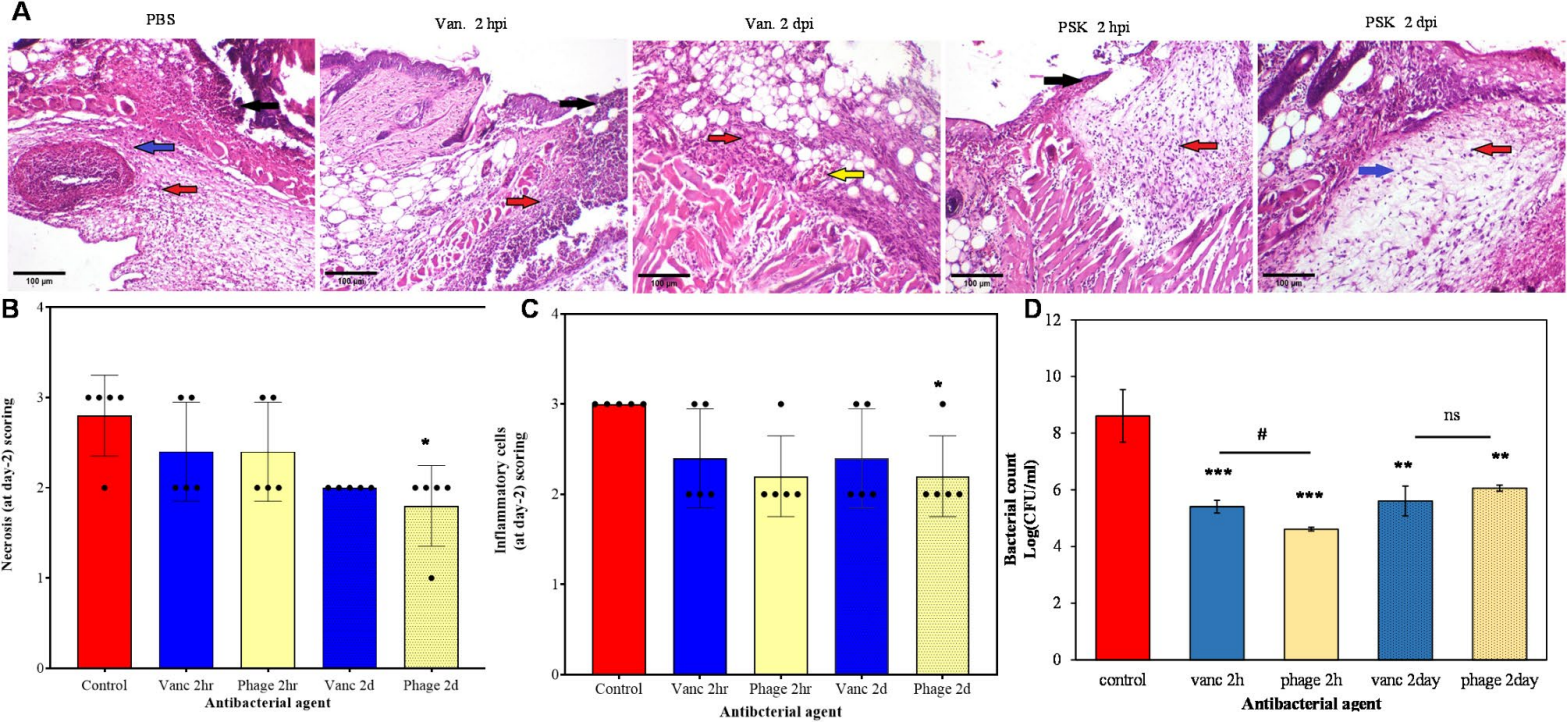
Representative photomicrographs exhibited Hematoxylin and Eosin (H&E)-stained skin Sect. (2 days) (scale bar, 100 μm, × 100). (**A**) Microscopic photographs of skin tissues obtained from control and treated groups. Control tissues display epidermal cells necrosis, intense inflammatory cells infiltration and marked edema. Vancomycin- treated group demonstrates inflammatory cells infiltration and congested blood vessel. Black arrow indicates necrosis of epidermal cells, red arrow indicates inflammatory cells, blue arrow indicates edema while yellow arrow indicates congested blood vessel. PSK-treated group (2 hpi) shows focal necrosis of epidermal epithelium and moderate inflammatory cells infiltration. PSK-treated group (2 dpi) shows inflammatory cells infiltration and edema. The statistical analysis of the histopathological scores of necrosis (**B**) and inflammatory cells infiltration (**C**). The data expressed as the median and interquartile range (p25-p75). Statistics were carried out by Kruskal Wallis test followed by Dunn test. (**D**) The antibacterial activity of either PSK or vancomycin after 2 hpi or dpi. *, *p* < 0.05; **, *p* < 0.01; ***, *p* < 0.001

Microscopical examination of the H&E-stained sections was performed at day 2 and day 8 timepoints to a have a deeper insight for the overall healing process. In consistency with wound closure experiment, control group in day 2 showed massive epithelial cell damage, infiltration of inflammatory cells and edema (Fig. 10). Early treatment with either vancomycin or PSK showed a slight reduction in necrosis and inflammatory cells infiltration (Fig. 10A, B and C). In addition, they showed significant antibacterial activities of 2.99 and 3.99 log-unit reductions in bacterial count respectively (Fig. 10D).

Whereas vancomycin-delayed treatment displayed comparable scores of necrosis and cells infiltration to the early treatment, PSK showed a significantly lower necrosis and fewer infiltrated inflammatory cells (Fig. 10A, B, C). Nonetheless, both agents showed a comparable (*p* > 0.05) bacterial count reductions of 5.6 ± 0.52 and 6.05 ± 0.10 log units for vancomycin and PSK respectively (Fig. 10D).

**Fig. 11:**
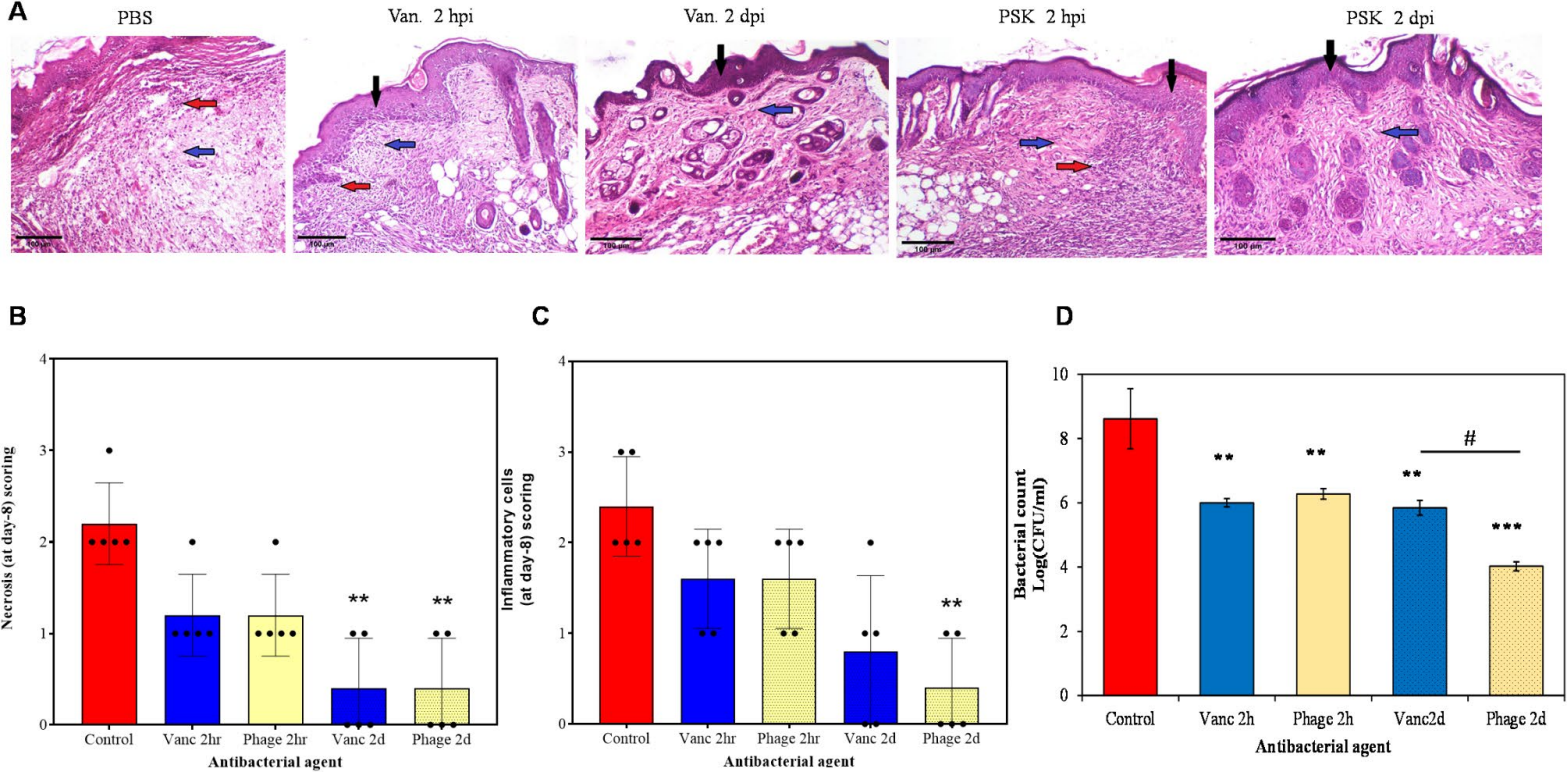
Representative photomicrographs exhibited H & E-stained skin Sect. (8 days) (scale bar, 100 μm, × 100). (**A**) Microscopic photographs of skin tissues obtained from control and treated groups after 8 days. The untreated group shows intense inflammatory cells infiltration and marked edema. PSK- and vancomycin- treated groups (2 hpi) demonstrate re-epithelialization, inflammatory cells infiltration and granulation tissue formation. The delayed treatment with both agents (2dpi) showing re-epithelialization and good granulation tissue formation. Black arrows indicate re-epithelialization; red arrows indicate inflammatory cells; blue arrow indicate granulation tissue. The statistical analysis of the histopathological scores of necrosis (**B**), inflammatory cells infiltration (**C**). The data expressed as the median and interquartile range (p25-p75). Statistics were carried out by Kruskal Wallis test followed by Dunn test. (**D**) The antibacterial activity of either PSK or vancomycin after 2 hpi or dpi. *, *p* < 0.05; **, *p* < 0.01; ***, *p* < 0.001

Another microscopical examination was also performed at the end of the experiment (day 8) to monitor the progress of wound healing process (Fig. 11). Again, control group tissues showed intense necrosis and inflammatory cells infiltration that were slightly improved upon early treatment with vancomycin or PSK or delayed treatment with vancomycin. Nonetheless, the outcome was outspoken for the group treated with PSK (2 dpi) with a significantly lower necrosis and inflammatory cells (*p* < 0.05, Fig. 11A, B, C), explaining the complete healing observed with wound closure experiment. This is further mirrored by the significantly higher granulation and re-epithelization scores observed in tissues from the group treated with PSK (2 dpi, Figure S3 in the supplementary material).

To assess wound repair at a microscopic level, we used Masson’s trichrome staining to monitor collagen deposition in the wound (Supplementary Figure S4A). Both PSK and vancomycin showed an improved wound repair, observed as the larger stained area, compared to the untreated group (Supplementary Figure S3). Interestingly, both agents exhibited a higher collagen deposition upon delayed interventions. For instance, vancomycin and PSK demonstrated collagen deposition of 43.7 and 56.5% after delayed treatment compared to 34 and 33.9% after early treatment respectively (Supplementary Figure S4). This is in the same line with the superior antibacterial performance exhibited by PSK following delayed treatment, 4.58 log unit reduction in bacterial count compared with maximum antibacterial activity of up to 2.6 log units with the other treatment options (Fig. 11D). These results highlight PSK as a potent therapeutic alternative, outperforming vancomycin, particularly in delayed treatment scenarios.

## Discussion

Treatment of MRSA infections is challenging, especially skin infections that show resistance to topically applied antibiotics (e.g., fusidic acid, gentamicin, mupirocin). These infections often necessitate the use of systemic antibiotics, like vancomycin and linezolid, which can expose patients to serious side effects [53, 54]. Our initial assessment of four wound *S. aureus* isolates revealed *S. aureus* SK1 as an MRSA strain based on its resistance to oxacillin and cefoxitin (Table 1). The strain also exhibits resistance to standard topical antibiotics (fusidic acid, sulfadiazine, gentamicin), which are routinely prescribed for wound infections. Consequently, we used the sewage samples collected from the hospital where we recovered S. aureus SK1 to isolate an effective lytic phage. Successfully, one phage was isolated (PSK) that showed clear plaques with podovirus morphology (Fig. 1).

PSK displayed infectivity against 4 out of the 10 tested strains (40%), indicating a relatively narrow host spectrum (Table 1). Nonetheless, its activity against challenging MRSA and VRSA strains encouraged us to pursue subsequent investigation (Table 1). This is in accordance with *S. aureus* phages showing comparable spectra covering up to 50% of the tested strains [55, 56]. Nonetheless, phages displaying broader spectra, reaching up to 85% of the test strains, have also been reported [57]. Host spectrum is determined by the specific interaction of the RBR, located at the tip of the phage tail fiber or spike, with their complementary receptors exposed on bacterial host surfaces (capsule, flagella, lipopolysaccharide) [12]. The variation in RBP sequences accounts for the varying host spectrums observed among different phages. This aligns with the dissimilarity observed within RBP (*orf8*) and tail fiber (*orf6*) encoding genes, particularly C-termini responsible for interaction with bacterial host, explaining the different host ranges amongst the homologous phages (Supplementary Figure S2). On the other side, BLASTn analysis revealed high nucleotide sequence similarity (up to 94% with Staphylococcus phage Simurgh, OQ302593.1) and intergenomic identity (up to 91.8% with Staphylococcus phage 351Saur083PP, OR062948.1), exceeding the genus classification cut-off value (70%). This suggests PSK as member of *Rosenblumvirus* genus under *Caudoviricetes* class. Nevertheless, none of the closest relatives showed nucleotide homology higher than 95%, proposing PSK as a new species of the genus *Rosenblumvirus* to the International Committee on Taxonomy of Viruses (ICTV) subcommittee chair [45].

Virulent phages are preferred for clinical use, not only because of their potent lytic activity against their bacterial host but also due to the limited propensity to disseminate virulence and antibiotic resistance genes [58]. Consequently, we performed sequential in silico and in vitro experiments to verify the lytic nature of PSK and hence, its suitability for clinical applications. In silico analysis revealed the absence of any potential virulence or antibiotic resistance genes. Moreover, PSK virulence was further confirmed using the Phage-AI online tool that has excluded lysogen-related genes (e.g., integrases and excisionase) [59]. Then, we extended our investigation to validate the capability of PSK to perform a complete lytic cycle using a one-step growth curve. PSK exhibited adsorption of > 75% of phage particles within 5 min, producing 123 PFU/infected cell after completing the cycle (Fig. 2A and B). Moreover, PSK has retained its infectivity up to 60 °C and over pH range of 4–11 (Fig. 2C&D). These findings indicate that the PSK is a lytic phage with acceptable environmental stability, making it well-suited for production and purification processes, ensuring its efficacy in clinical applications. For instance, PSK thermal stability at room temperature allows easy transport and storage without the need for refrigeration. This is particularly important in low-resource environments or during emergency outbreaks. Moreover, its thermal stability at higher temperatures could make PSK more adaptable to a wide range of geographical locations, including tropical and subtropical regions where bacterial resistance is often a concern. In addition, its wide pH stability makes it suitable for applications in body compartments with varying pH values, urine (5–6).

Additionally, we assessed the antibacterial potential of PSK in vitro against the MRSA SK1 strain. PSK showed ability to control the growth of the MRSA SK1 strain at different MOIs 10^2^- 10^− 4^ (Fig. 6A). Nevertheless, resistant mutants were detected at the end of the experiment (30 h) with a calculated frequency of 2.47 × 10^− 6^ ± 1.19 × 10^− 6^ CFU/mL (Fig. 6B). Emergence of BIM have also been reported against *S. aureus* [59, 60], *Klebsiella pneumoniae*,* Pseudomonas aeruginosa* [61] and *Acinetobacter baumannii* [24]. Phage resistance could occur as a result of different underlying mechanisms, including infection receptor modification, expression of phage genome degrading enzymes, CRISPR/Cas system in addition to bacteriophage exclusion system (BREX) that is working through retarding phage replication (reviewed in [62]). Phage resistance is a common challenge encountered during phage therapy and can lead to treatment failure if no new effective phages are introduced. Nonetheless, phage resistance may lead to phenotypic traits with increased antibiotic sensitivity, low growth rates, and less virulence that could be easily cleared by the immune system [60, 61, 63–65]. Different strategies have been introduced to limit BIM emergence, including using phage cocktail with individual phages with different molecular targets and antibiotic combination. In the current study, combining PSK with oxacillin, fusidic acid or vancomycin has significantly reduced resistance frequency. However, no significant difference was observed between individual PSK-antibiotic combinations (Fig. 6B). Surprisingly, the lower frequency mutation could not be translated into a synergistic bacterial outcome, underlining a possible unoptimized factor (treatment timing or antibiotic concentrations). Several studies have proposed the multifactorial origin of the varied outcome of phage-antibiotic combination including phage kinetics, antibiotic mechanism of action or concentration [66, 67]. In our case, the relatively rapid kinetics of PSK, leading to lower bacterial growth rates, may account for the lack of a synergistic effect. This decrease in growth may render antibiotics ineffective, as they require actively growing bacteria to exert their effects. Yet, the exact mechanism warrants further investigation using different antibiotic concentrations and timing, which is a limitation of our study.

Alternatively, PSK was investigated as a potential alternative for vancomycin in the treatment of the MRSA-induced wound infection model. In early treatment, one dose of PSK showed a comparable therapeutic outcome to vancomycin (administered as four doses, Fig. 9). This suggests that PSK may offer a more efficient treatment option, potentially reducing the burden of frequent dosing associated with traditional antibiotics. Nonetheless, when mice were treated 2 days after infection with both agents, PSK showed better performance than vancomycin. This was evidenced by a more significant reduction in bacterial counts, a smaller wound diameter, decreased inflammatory cell infiltration, and increased collagen deposition. The superior performance of PSK in the delayed treatment scenario indicates its potential to not only inhibit bacterial growth but also to promote more effective healing responses.

## Conclusion

MRSA skin infections are challenging, especially with limited effective topical antibiotics, thus necessitating repeated doses of systemic antibiotics. PSK is a promising solution for those infections given its rapid kinetics, high stability along with its infectivity to VRSA and MRSA strains. The conducted in silico and in vitro investigation have verified its lytic nature and suitability for clinical evaluation. Noteworthy, a single dose of PSK showed a comparable outcome to four doses of vancomycin in the murine wound model. Nonetheless, its better performance in the late treatment scenario makes it a suitable alternative, particularly in cases where traditional antibiotics are less effective or require multiple doses.

## Electronic supplementary material

Below is the link to the electronic supplementary material.

Supplementary Material 1

## Data Availability

The nucleotide sequence of the Staphylococcus phage PSK was deposited in NCBI GenBank database under accession number PQ110032.
