# Supplementary material for: Characterization, genomic analysis and preclinical evaluation of the lytic Staphylococcus bacteriophage PSK against methicillin-resistant Staphylococcus aureus wound isolate
Source: Ann Clin Microbiol Antimicrob. 2025 Feb 28;24:17. doi: 10.1186/s12941-025-00783-x (PMC11871620; doi:10.1186/s12941-025-00783-x)
Supplement: Supplementary file 1 — Supplementary Material 1 [file 12941_2025_783_MOESM1_ESM.docx]

Table S1. Annotation of the PSK genome and functional analysis of the detected *orf*s using BLASTx analysis against non-redundant database and search for conserved domains. Predicted function was assigned according to the best hit (highest identity and coverage). TR corresponds to terminal repeats

| *orf* | Start  (nt) | End  (nt) | Length (nt) | Start codon | aa | Predicted function | Closest hit | Identity%  (coverage %) | Accession number | Conserved domain  (E-value) |
| --- | --- | --- | --- | --- | --- | --- | --- | --- | --- | --- |
| TR | 6 | 249 | 243 |  |  | Terminal repeats |  |  |  |  |
| 1 | 326 | 733 | 408 | ATG | 140 | Hypothetical protein | Hypothetical protein phiAGO19_20 [Staphylococcus phage vB_SauP_phiAGO1.9] | 91.94  (91) | AUS03387.1 | PHA01819  (2.39e-14) |
| 2 | 748 | 930 | 183 | ATG | 60 | Hypothetical protein | Hypothetical protein SAP2_gp19 [Staphylococcus phage SAP-2] | 98.31  (96) | YP_001491544.1 | PHA01079  (1.29e-29) |
| 3 | 937 | 2163 | 1227 | ATG | 408 | Major head protein | major head protein [Staphylococcus phage PSa3] | 98.53  (99) | YP_009792353.1 | PHA01075  (0) |
| 4 | 2179 | 3162 | 984 | ATG | 327 | upper collar connector | upper collar connector [Staphylococcus phage GRCS] | 99.69  (99) | YP_009004308.1 | Phage_connector superfamily  (4.19e-04) |
| 5 | 3155 | 3910 | 756 | ATG | 251 | Lower collar protein | adaptor Ad4 [Staphylococcus phage PSa3] | 97.21  (99) | YP_009792351.1 | PHA01077  (5.85e-178) |
| 6 | 3924 | 5846 | 1923 | ATG | 640 | Minor tail protein | minor tail protein [Staphylococcus phage S24-1] | 87.52  (99) | YP_004957431.1 |  |
| 7 | 5858 | 6607 | 750 | ATG | 249 | Endolysin | endolysin [Staphylococcus phage Huma] | 95.58  (99) | WDS60848.1 | CHAP domain (4-134)  SH3-5 (164-228; 1.92e-07) |
| 8 | 6670 | 7566 | 897 | ATG | 298 | Rector binding protein | putative tail protein [Staphylococcus phage 351Saur083PP] | 90.6  (99) | WLY86760.1 |  |
| 9 | 7624 | 9387 | 1764 | ATG | 587 | Major tail protein | tail protein [Staphylococcus phage PSa3] | 98.47  (99) | YP_009792347.1 | Caud_tail_N  (1.81e-45) |
| 10 | 9389 | 9811 | 423 | ATG | 140 | Holin | holin [Staphylococcus phage SLPW] | 97.86  (99) | YP_009278563.1 | Phage_holin_4_1  (2.28e-11) |
| 11 | 9786 | 11219 | 1434 | ATG | 477 | Structural lysin | tail tip lysin [Staphylococcus phage 351Saur083PP] | 98.32  (99) | WLY86757.1 | CHAP domain  (0) |
| 12 | 13617 | 11329 | 2289 | ATG | 762 | DNA polymerase | DNA polymerase [Staphylococcus phage vB_SauP-V4SA02] | 96.84  (99) | WOZ17243.1 | DNA_pol_B_2  (7.64e-06) |
| 13 | 14879 | 13632 | 1248 | ATG | 415 | Packaging protein | terminase [Staphylococcus phage vB_SauP_EBHT] | 98.28  (99) | YP_010113612.1 | PHA01076  (0) |
| 14 | 15406 | 14927 | 480 | TTG | 159 | Hypothetical protein | hypothetical protein [Staphylococcus phage vB_SauR_SW21] | 98.11  (99) | WPF65106.1 | PHA01816  (7.92e-74) |
| 15 | 15985 | 15572 | 414 | ATG | 137 | Hypothetical protein | hypothetical protein GRCS_006 [Staphylococcus phage GRCS] | 68.84  (99) | YP_009004296.1 | PHA01814  (2.66e-40) |
| 16 | 16164 | 15988 | 177 | ATG | 58 | Hypothetical protein | hypothetical protein GRCS_005 [Staphylococcus phage GRCS] | 98.28  (98) | YP_009004295.1 | PHA01813  (5.64e-34) |
| 17 | 16581 | 16213 | 369 | ATG | 122 | DNA-binding protein | single stranded DNA-binding protein [Staphylococcus phage vB_SauR_SW21] | 98.38  (99) | WPF65109.1 | PHA01812  (8.18e-72) |
| 18 | 16841 | 16605 | 237 | ATG | 78 | Hypothetical protein | hypothetical protein [Staphylococcus phage vB_SauP_L1] | 96.15  (98) | WQY91100.1 | PHA01811  (1.98e-36) |
| 19 | 17161 | 16859 | 303 | ATG | 100 | Hypothetical protein | hypothetical protein KNV74_gp21 [Staphylococcus phage LSA2366] | 99  (99) | YP_010114672.1 | PHA01810  (1.57e-70) |
| TR | 17292 | 17535 | 244 |  |  | Terminal repeats |  |  |  |  |

**Table S2: The list of homologous phages.** This list was retrieved from NCBI based on BLASTn analysis of Staphylococcus phage vB_SauP_PSK nucleotide sequence.

| Bacteriophage name | Identity (%) | Coverage (%) | Accession |
| --- | --- | --- | --- |
| [Staphylococcus phage 351Saur083PP, complete genome](https://blast.ncbi.nlm.nih.gov/Blast.cgi#alnHdr_2566202050) | 93.69% | 95% | [OR062948.1](https://www.ncbi.nlm.nih.gov/nucleotide/OR062948.1?report=genbank&log$=nucltop&blast_rank=1&RID=AK098SH5016) |
| [Staphylococcus phage vB_SauR_SW21, complete genome](https://blast.ncbi.nlm.nih.gov/Blast.cgi#alnHdr_2621546118) | 93.82% | 95% | [OR683639.1](https://www.ncbi.nlm.nih.gov/nucleotide/OR683639.1?report=genbank&log$=nucltop&blast_rank=2&RID=AK098SH5016) |
| [Staphylococcus phage GRCS, complete genome](https://blast.ncbi.nlm.nih.gov/Blast.cgi#alnHdr_589892962) | 93.86% | 96% | [NC_023550.1](https://www.ncbi.nlm.nih.gov/nucleotide/NC_023550.1?report=genbank&log$=nucltop&blast_rank=3&RID=AK098SH5016) |
| [Staphylococcus phage vB_SauP_phiAGO1.9, complete genome](https://blast.ncbi.nlm.nih.gov/Blast.cgi#alnHdr_1345606604) | 89.27% | 97% | [MG766219.2](https://www.ncbi.nlm.nih.gov/nucleotide/MG766219.2?report=genbank&log$=nucltop&blast_rank=4&RID=AK098SH5016) |
| [Staphylococcus phage vB_SauP_phiAGO1.3, complete genome](https://blast.ncbi.nlm.nih.gov/Blast.cgi#alnHdr_1842010619) | 89.27% | 97% | [NC_047919.1](https://www.ncbi.nlm.nih.gov/nucleotide/NC_047919.1?report=genbank&log$=nucltop&blast_rank=5&RID=AK098SH5016) |
| [Staphylococcus phage vB_SauP_JS26 DNA, complete genome](https://blast.ncbi.nlm.nih.gov/Blast.cgi#alnHdr_2237838634) | 88.37% | 93% | [LC709172.1](https://www.ncbi.nlm.nih.gov/nucleotide/LC709172.1?report=genbank&log$=nucltop&blast_rank=6&RID=AK098SH5016) |
| [Staphylococcus phage SAPYZU_11, complete genome](https://blast.ncbi.nlm.nih.gov/Blast.cgi#alnHdr_2071749476) | 88.26% | 93% | [MW864250.1](https://www.ncbi.nlm.nih.gov/nucleotide/MW864250.1?report=genbank&log$=nucltop&blast_rank=7&RID=AK098SH5016) |
| [Staphylococcus phage vB_Sa_2868B2, complete genome](https://blast.ncbi.nlm.nih.gov/Blast.cgi#alnHdr_2504072047) | 88.23% | 93% | [OQ658779.1](https://www.ncbi.nlm.nih.gov/nucleotide/OQ658779.1?report=genbank&log$=nucltop&blast_rank=8&RID=AK098SH5016) |
| [Staphylococcus phage Simurgh, complete genome](https://blast.ncbi.nlm.nih.gov/Blast.cgi#alnHdr_2449454735) | 94.33% | 92% | [OQ302593.1](https://www.ncbi.nlm.nih.gov/nucleotide/OQ302593.1?report=genbank&log$=nucltop&blast_rank=9&RID=AK098SH5016) |
| [Staphylococcus phage Huma, complete genome](https://blast.ncbi.nlm.nih.gov/Blast.cgi#alnHdr_2449454715) | 94.28% | 92% | [OQ302592.1](https://www.ncbi.nlm.nih.gov/nucleotide/OQ302592.1?report=genbank&log$=nucltop&blast_rank=10&RID=AK098SH5016) |
| [Staphylococcus phage phiP68, complete genome](https://blast.ncbi.nlm.nih.gov/Blast.cgi#alnHdr_29565743) | 90.00% | 92% | [NC_004679.1](https://www.ncbi.nlm.nih.gov/nucleotide/NC_004679.1?report=genbank&log$=nucltop&blast_rank=11&RID=AK098SH5016) |
| [Staphylococcus aureus phage phiP68, complete genome](https://blast.ncbi.nlm.nih.gov/Blast.cgi#alnHdr_29378366) | 90.00% | 92% | [AF513033.1](https://www.ncbi.nlm.nih.gov/nucleotide/AF513033.1?report=genbank&log$=nucltop&blast_rank=12&RID=AK098SH5016) |
| [Staphylococcus phage SAP-2, complete genome](https://blast.ncbi.nlm.nih.gov/Blast.cgi#alnHdr_157738587) | 88.29% | 94% | [NC_009875.1](https://www.ncbi.nlm.nih.gov/nucleotide/NC_009875.1?report=genbank&log$=nucltop&blast_rank=13&RID=AK098SH5016) |
| [Staphylococcus phage vB_Sau-F2, complete genome](https://blast.ncbi.nlm.nih.gov/Blast.cgi#alnHdr_2756418933) | 93.99% | 94% | [PP910832.1](https://www.ncbi.nlm.nih.gov/nucleotide/PP910832.1?report=genbank&log$=nucltop&blast_rank=14&RID=AK098SH5016) |
| [Staphylococcus phage 66, complete genome](https://blast.ncbi.nlm.nih.gov/Blast.cgi#alnHdr_66395187) | 89.71% | 93% | [NC_007046.1](https://www.ncbi.nlm.nih.gov/nucleotide/NC_007046.1?report=genbank&log$=nucltop&blast_rank=15&RID=AK098SH5016) |
| [Bacteriophage 66, complete genome](https://blast.ncbi.nlm.nih.gov/Blast.cgi#alnHdr_62086017) | 89.71% | 93% | [AY954949.1](https://www.ncbi.nlm.nih.gov/nucleotide/AY954949.1?report=genbank&log$=nucltop&blast_rank=16&RID=AK098SH5016) |
| [Staphylococcus phage vB_SauP_EBHT, complete genome](https://blast.ncbi.nlm.nih.gov/Blast.cgi#alnHdr_2047458965) | 93.66% | 90% | [NC_055906.1](https://www.ncbi.nlm.nih.gov/nucleotide/NC_055906.1?report=genbank&log$=nucltop&blast_rank=17&RID=AK098SH5016) |
| [Staphylococcus phage SA4, partial genome](https://blast.ncbi.nlm.nih.gov/Blast.cgi#alnHdr_1241135094) | 88.38% | 59% | [MF001367.1](https://www.ncbi.nlm.nih.gov/nucleotide/MF001367.1?report=genbank&log$=nucltop&blast_rank=18&RID=AK098SH5016) |
| [Staphylococcus phage vB_SauP-V4SA02, complete genome](https://blast.ncbi.nlm.nih.gov/Blast.cgi#alnHdr_2618404244) | 90.30% | 89% | [OR602701.1](https://www.ncbi.nlm.nih.gov/nucleotide/OR602701.1?report=genbank&log$=nucltop&blast_rank=19&RID=AK098SH5016) |
| [Staphylococcus phage S13' DNA, complete genome](https://blast.ncbi.nlm.nih.gov/Blast.cgi#alnHdr_365189246) | 89.29% | 92% | [AB626963.1](https://www.ncbi.nlm.nih.gov/nucleotide/AB626963.1?report=genbank&log$=nucltop&blast_rank=20&RID=AK098SH5016) |
| [Staphylococcus phage phi44AHJD, complete genome](https://blast.ncbi.nlm.nih.gov/Blast.cgi#alnHdr_29565721) | 90.29% | 90% | [NC_004678.1](https://www.ncbi.nlm.nih.gov/nucleotide/NC_004678.1?report=genbank&log$=nucltop&blast_rank=21&RID=AK098SH5016) |
| [Staphylococcus phage SAPYZU_16, complete genome](https://blast.ncbi.nlm.nih.gov/Blast.cgi#alnHdr_2071749499) | 88.85% | 94% | [MW864251.1](https://www.ncbi.nlm.nih.gov/nucleotide/MW864251.1?report=genbank&log$=nucltop&blast_rank=22&RID=AK098SH5016) |
| [Staphylococcus phage Pabna, complete genome](https://blast.ncbi.nlm.nih.gov/Blast.cgi#alnHdr_1842029548) | 89.04% | 92% | [NC_048107.1](https://www.ncbi.nlm.nih.gov/nucleotide/NC_048107.1?report=genbank&log$=nucltop&blast_rank=23&RID=AK098SH5016) |
| [Staphylococcus phage S24-1, complete genome](https://blast.ncbi.nlm.nih.gov/Blast.cgi#alnHdr_371671319) | 89.02% | 95% | [NC_016565.1](https://www.ncbi.nlm.nih.gov/nucleotide/NC_016565.1?report=genbank&log$=nucltop&blast_rank=24&RID=AK098SH5016) |
| [Staphylococcus phage S24-1 DNA, complete genome](https://blast.ncbi.nlm.nih.gov/Blast.cgi#alnHdr_365189224) | 89.02% | 95% | [AB626962.1](https://www.ncbi.nlm.nih.gov/nucleotide/AB626962.1?report=genbank&log$=nucltop&blast_rank=25&RID=AK098SH5016) |
| [Staphylococcus phage vB_SauP_ASUmrsa123, complete genome](https://blast.ncbi.nlm.nih.gov/Blast.cgi#alnHdr_2560308780) | 88.78% | 96% | [OR259390.1](https://www.ncbi.nlm.nih.gov/nucleotide/OR259390.1?report=genbank&log$=nucltop&blast_rank=26&RID=AK098SH5016) |
| [Staphylococcus phage BP39, complete genome](https://blast.ncbi.nlm.nih.gov/Blast.cgi#alnHdr_695256149) | 88.54% | 98% | [KM366100.1](https://www.ncbi.nlm.nih.gov/nucleotide/KM366100.1?report=genbank&log$=nucltop&blast_rank=27&RID=AK098SH5016) |
| [Staphylococcus phage BP39, complete genome](https://blast.ncbi.nlm.nih.gov/Blast.cgi#alnHdr_1070100071) | 88.54% | 98% | [NC_031046.1](https://www.ncbi.nlm.nih.gov/nucleotide/NC_031046.1?report=genbank&log$=nucltop&blast_rank=28&RID=AK098SH5016) |
| [Staphylococcus phage Portland, complete genome](https://blast.ncbi.nlm.nih.gov/Blast.cgi#alnHdr_2047449371) | 88.13% | 90% | [NC_055814.1](https://www.ncbi.nlm.nih.gov/nucleotide/NC_055814.1?report=genbank&log$=nucltop&blast_rank=29&RID=AK098SH5016) |
| [Staphylococcus phage vB_SauP-436A1, complete genome](https://blast.ncbi.nlm.nih.gov/Blast.cgi#alnHdr_1720630513) | 88.20% | 92% | [MN150710.1](https://www.ncbi.nlm.nih.gov/nucleotide/MN150710.1?report=genbank&log$=nucltop&blast_rank=30&RID=AK098SH5016) |
| [Staphylococcus phage SCH1, complete genome](https://blast.ncbi.nlm.nih.gov/Blast.cgi#alnHdr_1841999980) | 88.20% | 92% | [NC_047788.1](https://www.ncbi.nlm.nih.gov/nucleotide/NC_047788.1?report=genbank&log$=nucltop&blast_rank=31&RID=AK098SH5016) |
| [Staphylococcus phage SCH111, complete genome](https://blast.ncbi.nlm.nih.gov/Blast.cgi#alnHdr_1103772812) | 88.19% | 92% | [KY000085.1](https://www.ncbi.nlm.nih.gov/nucleotide/KY000085.1?report=genbank&log$=nucltop&blast_rank=32&RID=AK098SH5016) |
| [Staphylococcus phage TSP, complete genome](https://blast.ncbi.nlm.nih.gov/Blast.cgi#alnHdr_1953871622) | 88.07% | 90% | [MW286254.1](https://www.ncbi.nlm.nih.gov/nucleotide/MW286254.1?report=genbank&log$=nucltop&blast_rank=33&RID=AK098SH5016) |
| [Staphylococcus phage vB_SauP_L1, complete genome](https://blast.ncbi.nlm.nih.gov/Blast.cgi#alnHdr_2642062848) | 88.12% | 93% | [OR944503.1](https://www.ncbi.nlm.nih.gov/nucleotide/OR944503.1?report=genbank&log$=nucltop&blast_rank=34&RID=AK098SH5016) |
| [Staphylococcus phage PSa3 complete sequence](https://blast.ncbi.nlm.nih.gov/Blast.cgi#alnHdr_1842005112) | 90.92% | 91% | [NC_047855.1](https://www.ncbi.nlm.nih.gov/nucleotide/NC_047855.1?report=genbank&log$=nucltop&blast_rank=35&RID=AK098SH5016) |
| [Staphylococcus phage vB_SA_STAP152, complete genome](https://blast.ncbi.nlm.nih.gov/Blast.cgi#alnHdr_2582126165) | 90.66% | 90% | [OR573959.1](https://www.ncbi.nlm.nih.gov/nucleotide/OR573959.1?report=genbank&log$=nucltop&blast_rank=36&RID=AK098SH5016) |
| [Staphylococcus phage 4086-1, complete genome](https://blast.ncbi.nlm.nih.gov/Blast.cgi#alnHdr_2705239367) | 90.47% | 92% | [PP541615.1](https://www.ncbi.nlm.nih.gov/nucleotide/PP541615.1?report=genbank&log$=nucltop&blast_rank=37&RID=AK098SH5016) |
| [Staphylococcus phage vB_SauR_SW25, complete genome](https://blast.ncbi.nlm.nih.gov/Blast.cgi#alnHdr_2664202472) | 90.55% | 92% | [PP135470.1](https://www.ncbi.nlm.nih.gov/nucleotide/PP135470.1?report=genbank&log$=nucltop&blast_rank=38&RID=AK098SH5016) |
| [Staphylococcus phage CSA13, complete genome](https://blast.ncbi.nlm.nih.gov/Blast.cgi#alnHdr_1842033044) | 90.19% | 94% | [NC_048159.1](https://www.ncbi.nlm.nih.gov/nucleotide/NC_048159.1?report=genbank&log$=nucltop&blast_rank=39&RID=AK098SH5016) |
| [Staphylococcus phage JPL-50, complete genome](https://blast.ncbi.nlm.nih.gov/Blast.cgi#alnHdr_2438087456) | 89.52% | 90% | [NC_070878.1](https://www.ncbi.nlm.nih.gov/nucleotide/NC_070878.1?report=genbank&log$=nucltop&blast_rank=40&RID=AK098SH5016) |
| [Staphylococcus phage SLPW, complete genome](https://blast.ncbi.nlm.nih.gov/Blast.cgi#alnHdr_1033016334) | 89.32% | 88% | [KU992911.1](https://www.ncbi.nlm.nih.gov/nucleotide/KU992911.1?report=genbank&log$=nucltop&blast_rank=41&RID=AK098SH5016) |
| [Staphylococcus phage SLPW, complete genome](https://blast.ncbi.nlm.nih.gov/Blast.cgi#alnHdr_1070096627) | 89.32% | 88% | [NC_031008.1](https://www.ncbi.nlm.nih.gov/nucleotide/NC_031008.1?report=genbank&log$=nucltop&blast_rank=42&RID=AK098SH5016) |
| [Staphylococcus phage LSA2366, complete genome](https://blast.ncbi.nlm.nih.gov/Blast.cgi#alnHdr_2047460022) | 89.23% | 91% | [NC_055916.1](https://www.ncbi.nlm.nih.gov/nucleotide/NC_055916.1?report=genbank&log$=nucltop&blast_rank=43&RID=AK098SH5016) |
| [Staphylococcus phage STPX-6, complete genome](https://blast.ncbi.nlm.nih.gov/Blast.cgi#alnHdr_2722331033) | 89.01% | 90% | [PP723060.1](https://www.ncbi.nlm.nih.gov/nucleotide/PP723060.1?report=genbank&log$=nucltop&blast_rank=44&RID=AK098SH5016) |
| [Staphylococcus phage SA46-CTH2, complete genome](https://blast.ncbi.nlm.nih.gov/Blast.cgi#alnHdr_1675871996) | 84.93% | 94% | [MK764384.1](https://www.ncbi.nlm.nih.gov/nucleotide/MK764384.1?report=genbank&log$=nucltop&blast_rank=45&RID=AK098SH5016) |
| [Staphylococcus phage SA44-CTH7, complete genome](https://blast.ncbi.nlm.nih.gov/Blast.cgi#alnHdr_1735346998) | 84.92% | 94% | [MK903033.1](https://www.ncbi.nlm.nih.gov/nucleotide/MK903033.1?report=genbank&log$=nucltop&blast_rank=46&RID=AK098SH5016) |
| [Staphylococcus phage SA46-CL1, complete genome](https://blast.ncbi.nlm.nih.gov/Blast.cgi#alnHdr_2047448043) | 84.87% | 94% | [NC_055802.1](https://www.ncbi.nlm.nih.gov/nucleotide/NC_055802.1?report=genbank&log$=nucltop&blast_rank=47&RID=AK098SH5016) |
| [Staphylococcus phage SA46-CTH4, complete genome](https://blast.ncbi.nlm.nih.gov/Blast.cgi#alnHdr_1726258805) | 84.82% | 95% | [MK922548.1](https://www.ncbi.nlm.nih.gov/nucleotide/MK922548.1?report=genbank&log$=nucltop&blast_rank=48&RID=AK098SH5016) |
| [Staphylococcus phage SA30-CTH2, complete genome](https://blast.ncbi.nlm.nih.gov/Blast.cgi#alnHdr_1726258785) | 84.82% | 95% | [MK922547.1](https://www.ncbi.nlm.nih.gov/nucleotide/MK922547.1?report=genbank&log$=nucltop&blast_rank=49&RID=AK098SH5016) |
| [Staphylococcus phage SA03-CTH2, complete genome](https://blast.ncbi.nlm.nih.gov/Blast.cgi#alnHdr_1698230588) | 84.83% | 95% | [MK936475.1](https://www.ncbi.nlm.nih.gov/nucleotide/MK936475.1?report=genbank&log$=nucltop&blast_rank=50&RID=AK098SH5016) |
| [Staphylococcus phage SA1-CTA1, complete genome](https://blast.ncbi.nlm.nih.gov/Blast.cgi#alnHdr_1726258765) | 84.57% | 94% | [MK922546.1](https://www.ncbi.nlm.nih.gov/nucleotide/MK922546.1?report=genbank&log$=nucltop&blast_rank=51&RID=AK098SH5016) |
| [Staphylococcus phage S-CoN_Ph35, complete genome](https://blast.ncbi.nlm.nih.gov/Blast.cgi#alnHdr_2582095479) | 93.69% | 95% | [OR062948.1](https://www.ncbi.nlm.nih.gov/nucleotide/OR062948.1?report=genbank&log$=nucltop&blast_rank=1&RID=AK098SH5016) |

**Table S3.** Putative promotors using the MEME tool (Bailey et al., 2009). The six promotors assigned as P1- P6 according to the order of their appearance on the lead strand. Consensus sequence represents the over-presented motif throughout PSK genome within intergenomic spaces.

| **Promotor** | **5' end nt position** | **Sequence** | **Consensus sequence** |
| --- | --- | --- | --- |
| P1 | 108 - 148 | GTGTGACAACAATTAACATTTATATAACAACTTACATATTC | 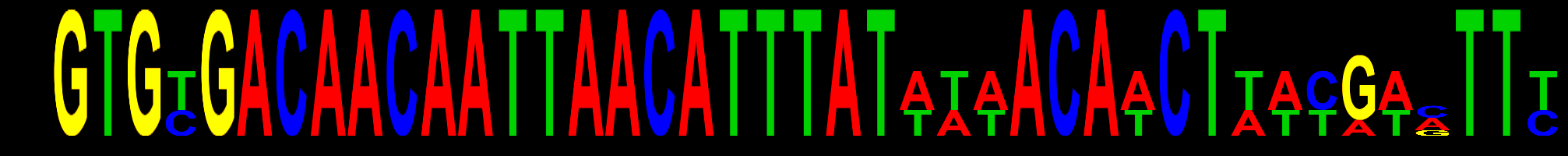 |
| P2 | 190 - 230 | GTGCGACAACAATTAACATTTATTATACATCTATTGACTTT |  |
| P3 | 17311 - 17351 | GTGCGACAACAATTAACATTTATTATACATCTATTGACTTT |  |
| P4 | 17393 - 17433 | GTGTGACAACAATTAACATTTATATAACAACTTACGTATTC |  |
| P5 | 17531 - 17571 | GTGTGACAACAATTAACATTTATATAACAACTTACGAGTTT |  |
| P6 | 2122 - 2170 | CAAGAAGTAACACCAAACCCAGAGGAAGAAGCAACAGAATAAAAAGGAG | 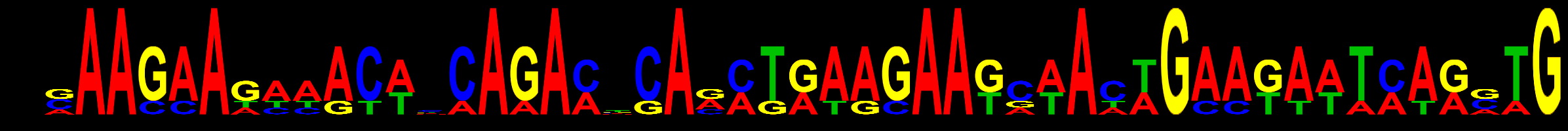 |

ho

**Table S4.** Putative rho-independent terminators in the PMK34 genome using the ARNold tool (Naville et al., 2011). Colors indicate sequence secondary structure: red indicates the loop sequence, whereas blue indicates the double stranded stem.

| **Terminator** | **Strand** | **Nt position** | **Sequence** | **ΔG** | **After gene** |
| --- | --- | --- | --- | --- | --- |
| T1 | - | 11308 | GACATCAAAAAGGCAACTGTTAAATAACAGTTGCCTTTTTCTTTTGAG | -12.70 | *orf13* |
| T2 | - | 16172 | ATACAATTTCATAGGGGTACTTATCCCCTATTTTTATGAGGT | -9.00 | *orf18* |


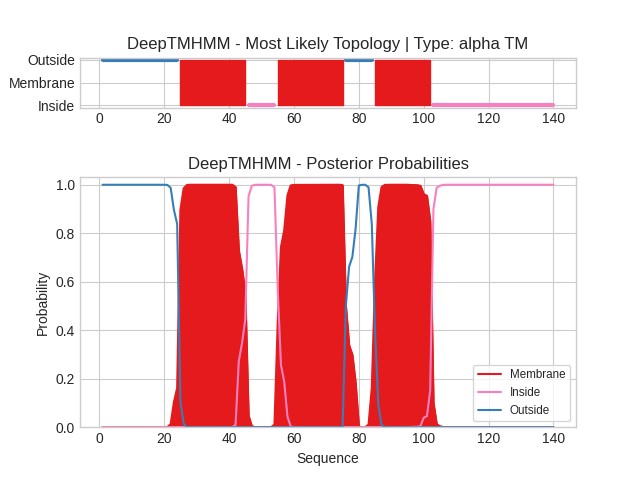


**Figure S1: Holin topolgy presentations using DeepTMHMM - Predictions.** The existance of each protein stretch either inside or outside the cytoplasmic membrance are represented as pink and blue line respectively. The domians with predicted transmembrane occurrence are displayed as red zones. The probalitity of occurrence of each stretch is presented on y-axis.

**Figure S2: Pairwise global alignment of PSK genome with the homologous phages’ genomes using DIGAlign tool.** BLASTx products of the PSK nucleotide sequence (PQ110032) were aligned with the top five BLASTn hits. The aligned sequences include phiAGO1.3 (NC_047919), SW21 (OR683639.1), 083PP (OR062948.1), Simurgh (OQ302593.1), GRCS (NC_023550.1), and Huma (OQ302592.1). The similarity level is depicted by the legend in the top-left corner. The left-handed panels represent the pairwise dot-plot of the full PSK genome with its phage genome counterpart. The vertical red box represents the receptor binding proteins (RBR) of the aligned phages.

**Figure S3: Assessment of wound healing process amongst different mice groups at day 8.** A) Granulation and B) re-epithelization scores of the obtained skin tissues obtained from untreated, PSK and vancomycin treated groups 2 hpi and 2 dpi. The data expressed as the median and interquartile range (p25-p75). Statistics were carried out by Kruskal Wallis test followed by Dunn test. ** Indicate a significance difference with control group at *p* < 0.01.

**Figure S4: Collagen deposition validation using Masson’s trichrome (MTC).** A) Representative photomicrographs of skin stained with MTC to identify collagen deposition (blue). The untreated group shows weak MTC stained collagen deposition. PSK or vancomycin treated groups (2hpi) exhibit a moderate increase in collagen. Delayed treatments show marked increased collagen which higher significance in case of PSK treatment (Scale bar, 100μm). (B) %Area of positive MTC staining. Data was expressed as mean ± standard deviation. Statistics were carried out by One-way ANOVA followed by Tukey's multiple comparisons test. Significance was considered at P < 0.0001. *Significantly different compared with control, # significantly different compared with vanc 2d.
